# Supplementary material for: Biomimetic supramolecular protein matrix restores structure and properties of human dental enamel
Source: Nat Commun. 2025 Nov 4;16:9434. doi: 10.1038/s41467-025-64982-y (PMC12586552; doi:10.1038/s41467-025-64982-y)
Supplement: Supplementary file 1 — Supplementary Information File [file 41467_2025_64982_MOESM1_ESM.pdf]

# Supplementary Information

# Biomimetic supramolecular protein matrix restores structure and properties of human dental enamel

Abshar Hasan[1,2,3,4], Andrey Chuvilin[5,6], Alexander Van Teijlingen[7], Helena Rouco[1,2], Christopher Parmenter[8], Federico Venturi[9], Michael Fay[8,9], Gabriele Greco[10,11], Nicola M. Pugno[11,12], Jan Ruben[13], Charlotte J.C. Edwards-Gayle[14], Benjamin Myers[15], Ingrid Dreveny[1], Nathan Cowieson[14], Adam Winter[16], Sara Gamea[17,18], X. Frank Walboomers[13], Tanvir Hussain[9], José Carlos Rodríguez-Cabello[19], Frankie Rawson[2,15], Tell Tuttle[7], Sherif Elsharkawy[17], Avijit Banerjee[17], Stefan Habelitz[20], Alvaro Mata[1,2,3,4,*]

* Corresponding author(s): a.mata@nottingham.ac.uk

**Content**


**1. Supplementary Discussions** **Page**

Supplementary Discussion 1: Rationale behind using 1% w/v ELR for preparing ELR fibrils. 5

Supplementary Discussion 2: Modulation of the ELR conformation within the fibrillar matrix. 6

Supplementary Discussion 3: Ca ions and crosslinking regulate the formation of supramolecular ELR structures in solution. 7

Supplementary Discussion 4: Use of nail varnish to demonstrate remineralization on aprismatic enamel. 8

Supplementary Discussion 5: Interplay between mineralization and degradation of the ELR matrix. 9

Supplementary Discussion 6: Characterization of chemical composition of remineralized enamel. 10

Supplementary Discussion 7: Remineralized enamel exhibit comparable crystal structure to native enamel. 11

Supplementary Discussion 8: Model explains difference in E and H values for remineralized enamel and dentine surfaces. 12

Supplementary Discussion 9: Effect of solvent composition, environmental conditions, and substrate on the secondary structure conformation of the ELR matrix. 14

Supplementary Discussion 10: Confirmation of the strong interfacial bonding (i.e., integration) between the newly formed enamel and native enamel/dentine. 15

Supplementary Discussion 11: Summary of the key mechanistic features of the ELR mediated remineralization of enamel. 16

**2. Supplementary Figures**

Supplementary Fig. 1: TEM and SEM imaging of the ELR fibrils. 17

Supplementary Fig. 2: Characterization of ELR-Ca interactions. 18

Supplementary Fig. 3: Secondary structure conformation of ELR in solution and in matrix at different Ca concentration. 19

Supplementary Fig. 4: Secondary structure composition of ELR coatings on enamel surface. 20

Supplementary Fig. 5: Quantification of secondary structure conformation and staining of β-sheet rich regions in crosslinked ELR membranes. 21

Supplementary Fig. 6: Ca ions and crosslinking regulate the formation of supramolecular ELR structures in solution. 22

Supplementary Fig. 7: Increasing the Ca concentration in ELR matrix increases mineralization. 23

Supplementary Fig. 8: X-ray scattering analysis. 24

Supplementary Fig. 9: Coarse-grained simulation confirms ELR filament formation in presence of $Ca^{2+}$ ions and their assembly into fibril. 25

Supplementary Fig. 10: Chemical analysis of mineralized ELR fibres. 26

Supplementary Fig. 11: Coarse-grained simulation showing detachment of a small ELR fragment from a- and c-axes of the fluorapatite crystal. 27

Supplementary Fig. 12: Uncontrolled mineralization on enamel and dentine surface in the absence of ELR matrix. 28

Supplementary Fig. 13: TEM image showing crystal growth in the presence of ELR matrix. 29

Supplementary Fig. 14: Optimization of ELR coating thicknesses on enamel surface. 30

Supplementary Fig. 15: Enamel remineralization using artificial saliva. 31

Supplementary Fig. 16: Fabrication of mineralized layer on prismatic and aprismatic enamel regions. 32
Supplementary Fig. 17: Tuneability of mineral layer thickness on dentine. 33
Supplementary Fig. 18: Uncontrolled mineralization on enamel and dentine surface in the absence of ELR matrix. 34
Supplementary Fig. 19: SEM and confocal imaging. 35
Supplementary Fig. 20: Atomic force microscopic (AFM) imaging of mineralized enamel section. 36
Supplementary Fig. 21: Acid etched and remineralized diazone region of enamel. 37
Supplementary Fig. 22: Remineralized parazone region of enamel. 38
Supplementary Fig. 23: High magnification SEM images confirm complete ELR degradation post elastase treatment. 39
Supplementary Fig. 24: Interplay between mineralization and degradation of the ELR matrices. 40
Supplementary Fig. 25: Specific wear rate and wear strength calculation for remineralized enamel. 41
Supplementary Fig. 26: Estimation of the crystal density and its co-relation with Wear strength (WS). 42
Supplementary Fig. 27: EDX and XRD analysis of remineralized enamel. 43
Supplementary Fig. 28: SEM images of remineralized parazone region of enamel. 44
Supplementary Fig. 29: EDX analysis confirms FAp formation on parazone region of enamel. 45
Supplementary Fig. 30: HAp formation using mineralization solution devoid of F- ions. 46
Supplementary Fig. 31: Integration between the mineralized layer and mineralized collagen fibrils (MCFs) at interface. 47
Supplementary Fig. 32: TEM-EDX mapping of the mineralized dentine surface. 48
Supplementary Fig. 33: Customized setup to simulate tooth brushing. 49
Supplementary Fig. 34: SEM images of acid-etched and remineralized prismatic enamel after indentation. 50
Supplementary Fig. 35: Setup to simulate tooth abrasion. 51
Supplementary Fig. 36: Surface mineralization protects dentine against acid attack. 52
Supplementary Fig. 37: Live/dead staining of human immortalised mesenchymal stem cells (hiMSCs). 53
Supplementary Fig. 38: Live/dead staining of mouse fibroblast 3T3 cells. 54
Supplementary Fig. 39: Live/dead staining of human umbilical vein endothelial cells (HUVECs). 55
Supplementary Fig. 40: Cell viability determination by MTS assay. 56
Supplementary Fig. 41: ELR matrix diffuses deep into the inter-crystal voids on enamel surface. 57
Supplementary Fig. 42: Uniform ELR coating over large and convoluted tooth area. 58
Supplementary Fig. 43: Ethanol/water system forms ELR fibrillar ensembles, recreates microstructure, and restores mechanical properties. 59
Supplementary Fig. 44: Effect of solvent concentration and crosslinker concentration on the secondary structure conformation of the ELR matrices. 60
Supplementary Fig. 45: Enamel remineralization under real oral environments. 61
Supplementary Fig. 46: Enamel remineralization using natural saliva. 62
Supplementary Fig. 47: ELR coating is highly stable against different chemical treatments. 63
Supplementary Fig. 48: ELR coating is highly stable against different chemical treatments. 64

Supplementary Fig. 49: Characterization of ELR coating after physical and chemical treatments. 65
Supplementary Fig. 50: SEM images of an ELR coated enamel. 66
Supplementary Fig. 51: Profilometry analysis of the ELR coated tooth enamel. 67
Supplementary Fig. 52: ELR coating Vs Duraphat® coating. 68

**3. Supplementary references** 69

## 1. Supplementary Discussions

**Supplementary Discussion 1: Rationale behind using 1% w/v ELR for preparing ELR fibrils.**
ELR fibrils were observed under both SEM and TEM by drying a drop of 1% w/v ELR solution containing 1.5 mM $Ca^{2+}$ ions and without HDI crosslinking. At this ELR concentration, individual ELR fibrils can be observed (**Fig. 1a, Supplementary Fig. 1a, b**). 5% w/v ELR concentration was too high to observe individual fibrils both under SEM and TEM. However, for investigating mineralization of the ELR fibrils under TEM, we used the standard 5% w/v ELR solution containing 1.5 mM $Ca^{2+}$ ions and simultaneous crosslinking using 0.56% v/v HDI as used when creating ELR coatings on enamel and dentine surfaces.

**Supplementary Discussion 2: Modulation of the ELR conformation within the fibrillar matrix.**
We investigated the possibility to modulate the formation and conformation of these ELR fibrils as a mechanism to tune mineralization. First, using FTIR and a 5% w/v ELR solution, we found that increasing $Ca^{2+}$ concentration from 0 to 1.5 mM increased β-conformations (*i.e.,* β-sheet, β-turn) from 10% to 25%, respectively (**Supplementary Fig. 3a**). Interestingly, further increase in $Ca^{2+}$ concentration did not lead to changes in ELR secondary structure, suggesting saturation of all the $Ca^{2+}$ binding sites in the ELR molecules. Second, we tested the use of crosslinking and drying of the ELR solution as an additional mechanism to tailor fibril formation. FTIR analysis of 5% w/v ELR solution (without $Ca^{2+}$) in the presence of 0.56% v/v HDI[1] revealed that β-conformations increased from ~10 to 52% upon simultaneous drying and crosslinking. As expected, combining the incorporation of $Ca^{2+}$ ions and the drying/cross-linking steps led to higher increases (up to ~80%) in β-conformations (**Supplementary Fig. 3b, c**) and in quantity of fibrils (**Supplementary Fig. 5b, c**). These results demonstrate the possibility to use $Ca^{2+}$ ions during the ELR matrix fabrication to trigger ELR disorder-to-order transitions and engineer ELR matrices with tuneable levels of β-conformations and fibril formation, while increasing the number of potential nucleation points for mineralization (**Supplementary Fig. 7**).

**Supplementary Discussion 3: Ca ions and crosslinking regulate the formation of supramolecular ELR structures in solution.**

The results demonstrate that ELR molecules in the presence of Ca ions in a DMF/DMSO solvent mixture exhibit an increase in β-conformations (**Supplementary Fig. 3a** and **Supplementary discussion 2**), suggesting assembly into fibre-like supramolecular structures as previously reported[2]. To confirm this, we performed dynamic light scattering (DLS) and FTIR analyses in a biocompatible ethanol/water (9/1) mixture, chosen over DMF/DMSO to align with the translational focus of our study. The ELR solution (1 mg/mL) without Ca ions exhibited structures with hydrodynamic radius ($R_h$) of 2.3 nm (**Supplementary Fig. 6b**), which is similar to the size of monomers reported for similar sized proteins (~33 kDa)[3]. However, addition of 1.5 mM Ca ions to the above ELR solution resulted in the formation of ELR structures with increased size ($R_h$ = 8.7 nm) and amounts of β-sheet conformation (36.7% compared to 28.9% without Ca) (**Supplementary Fig. 6d, e**). Size distribution analysis of these structures revealed the formation of spherical shaped ELR aggregates or oligomers as reported for other proteins[4,5]. Furthermore, addition of a small amount of glutaraldehyde crosslinker (0.1%) to the ELR solution containing Ca ions resulted in the formation of additional, larger structures (ranging from 300 nm to 2000 nm) with a further increased β-sheet conformation (48.2%) (**Supplementary Fig. 6f, g**). These results corroborate the critical role of Ca ions and crosslinking in the formation of supramolecular ELR structures in solution.

**Supplementary Discussion 4: Use of nail varnish to demonstrate remineralization on aprismatic enamel.**

To distinctly show the remineralized layer on aprismatic enamel, we covered a portion of the enamel surface with acid resistant nail varnish and the remaining portion with the ELR coating. After remineralization, the ELR coating and nail varnish were removed using enzymatic elastase digestion and acetone treatment, respectively. The step (black shadow) observed between the remineralized layer and the native enamel in the left-most image of **Fig. 2d** corresponds to this interface where the portion covered with nail varnish meets the portion that was covered with the ELR coating.

**Supplementary Discussion 5: Interplay between mineralization and degradation of the ELR matrix.** We have previously described how the ELR molecules assemble into a dense matrix with an ELR conformation that enables the growth of highly organised mineralized structures[1]. This study also demonstrated that this densely packed and crosslinked ELR matrix remains present during the mineralization process and can be completely degraded using elastase enzymes, demonstrating that the ELR matrix is both necessary to trigger organized mineralization and biodegradable.

To further investigate this mineralization/degradation process, we first conducted swelling experiments by exposing the ELR matrix to a mineralizing pH = 6 condition, which revealed a 22% swelling in this condition (**Supplementary Fig. 24a**). We hypothesized that this swelling would enhance ELR matrix degradation and thus we performed degradation tests by exposing the ELR matrix to a mineralization solution in the absence or presence of salivary enzymes for 3 weeks. The results confirmed that the ELR matrix degraded by 14.8 ± 0.4 wt% and 18.5 ± 0.2 wt% in the absence and presence of salivary enzymes, respectively (**Supplementary Fig. 24b**).

We then conducted a thermogravimetric analysis (TGA) to assess differences in the degradation of the organic ELR matrix before and after mineralization. The results further confirmed a loss of ~15 wt% of the ELR matrix during the period of mineralization (**Supplementary Fig. 24c**), which corresponds to the results from the degradation experiment (**Supplementary Fig. 24b**). In addition, given this swelling and degradation of the matrix as well as the critical role of the secondary structure of the ELR for mineralization, we then conducted FTIR experiments to assess changes during the mineralization process. The results revealed that the secondary structure conformation of the ELR did not change significantly during the mineralization process (**Supplementary Fig. 24d**).

Overall, these results demonstrate that the ELR matrix undergoes slow degradation but remains both stable and functional (i.e., maintaining its secondary structure conformation) during the distinctive epitaxial and organized mineralization in both artificial saliva (**Supplementary Fig. 15, 45**) and natural saliva (**Fig. 6j, k,** and **Supplementary Fig. 46**) conditions.

**Supplementary Discussion 6: Characterization of chemical composition of remineralized enamel.** Energy-dispersive X-ray spectroscopy (EDX) analysis was used to characterize the chemical composition of the remineralized enamel, confirming the presence of calcium, phosphorus, and fluorine (**Supplementary Fig. 27a, 29a**) at atomic Ca/P ratio similar to those of apatite crystals. This result suggested the formation of fluoride-substituted apatite (fluorapatite, FAp) nanocrystals[1] in contrast to carbonate-substituted hydroxyapatite (carbonated hydroxyapatite, CHAp) in native enamel[6]. Furthermore, FTIR analysis of the remineralized enamel revealed the presence of the typical phosphate peaks at 604 and 565 $cm^{-1}$ ($v_4(PO_4)$) and 1035 $cm^{-1}$ $v_3(PO_4)$ (**Supplementary Fig. 29b**), indicating apatite formation[7]. This formation was confirmed by X-ray diffraction (XRD), revealing diffraction peaks and their relative intensities comparable to those of native enamel (**Supplementary Fig. 29c**) and standard fluorapatite (JCPDF #73-1727) (**Supplementary Fig. 27b**). These results indicate that our ELR matrix can remineralize enamel with similar chemical composition as native enamel tissue. However, CHAp nanocrystals are grown when using the ELR matrix and a mineralization solution devoid of $F^-$ ions (**Supplementary Fig. 30**).

**Supplementary Discussion 7: Remineralized enamel exhibit comparable crystal structure to native enamel.**

Our TEM results (**Fig. 4h, j**) demonstrate a slight variation in the crystal structure in parazone region which could be due to sectioning artifacts during FIB processing, thus exaggerating an apparent difference between the remineralized layer and the native tissue. However, in conjugation with other techniques such as SEM (**Fig. 3e, and Supplementary Fig. 22, 23, 27, 28**), FFT (**insets in Fig. 4i**), SAED (**insets in Fig. 4j**), and XRD (**Supplementary Fig. 27b**), this variation appears to be negligible. In addition, the typical hexagonal morphology of the apatite nanocrystals in the remineralized layer (**rightmost image in Fig. 2d, Fig. 3d and e, Supplementary Fig. 23, 43c, d**) confirmed that their crystal structure is similar to native enamel crystals. Furthermore, FTIR and XRD results confirmed that the remineralized layer comprised of CaP mineral and exhibited an apatite phase similar to native enamel (**Supplementary Fig. 27b, 29b, c**). Beyond this individual nanocrystal structure, the ELR platform enables epitaxial and organized growth of densely packed apatite nanocrystals (**Supplementary Fig. 26**), which leads to recreation of the hierarchical structure of native prismatic and aprismatic enamel (**Fig. 2d and g, Fig. 3d and e**) and, consequently, restoration of the mechanical (**Fig. 3f**) and tribological properties (**Fig. 3g, h**). Overall, our results demonstrate that the remineralized layer exhibit crystal structure comparable to native enamel.

**Supplementary Discussion 8: Model explains difference in E and H values for remineralized enamel and dentine surfaces.**

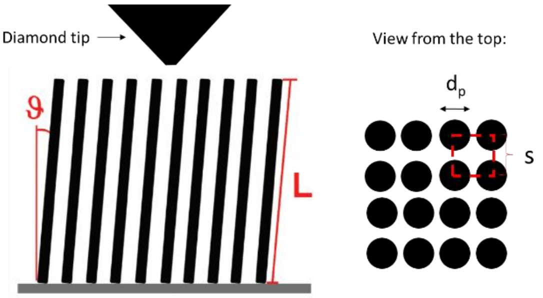


Based on the model proposed by Pavese et al.[8], we aimed to explain the difference in the measured hardness (H) and Young's modulus (E) for remineralized enamel and dentin samples. When indenting samples with vertically mineralized crystals (i.e., in diazone), the diamond tip of the indenter will compress a bundles of apatite nanocrystals as depicted in the above image.
We assigned the length of these apatite nanocrystals as 'L', diameter '$d_p$', organized at an inclination of 'ϑ', and separated from each other with a distance 's'.

During indentation, the machine measures an apparent modulus (E') and hardness (H') which can be related to the real E and H of the nanocrystals using expression reported by Pavese et al.[8] i.e.,

$$E' = \frac{E\varphi}{1+\frac{tan^2(\theta)\lambda^2}{3}} \qquad (1)$$

$$H' = c\,\frac{\pi H\varphi}{1+4\lambda^2} \qquad (2)$$

Where $1-\varphi$ is the fraction of empty spaces, $\lambda$ is an a-dimensional constant given by $\lambda = 4L/d_p$, and $c = \frac{H}{\sigma_P}$ where $\sigma_P$ is the stress at elastic instability of the pillars.

In case of dentine samples, there are only vertically mineralized crystals whereas enamel contains both vertical (diazone) and horizontal (parazone) crystals. This implies that when indenting an enamel samples, the values of H and E must be averaged considering the fraction of vertical and horizontal crystals, $\phi_v$ and $\phi_h$ respectively (with $\phi_h + \phi_v = a$), as well as the fraction of empty spaces *1-a*.

Therefore, for enamel:

$$E'_e = \frac{E\varphi_v}{1+\frac{tg^2(\theta)\lambda^2}{3}} + E\varphi_h \qquad (3)$$

$$H'_e = c\,\frac{\pi H\varphi_v}{1+4\lambda^2} + H\varphi_h \qquad (4)$$

and for dentine:

$$E'_d = \frac{Ea}{1+\frac{tg^2(\theta)\lambda^2}{3}} \qquad (5)$$

$$H'_d = c\,\frac{\pi Ha}{1+4\lambda^2} \qquad (6)$$

With these expressions, we can calculate the differences (here expressed as ratios) between the values measured for enamel (*e*) and those for dentine (*d*). These are:

$$\frac{E'_e}{E'_d} = 1 + \frac{\varphi_h}{a}\frac{tg^2\,\theta\,\lambda^2}{3} \qquad (7)$$

$$\frac{H'_e}{H'_d} = 1 + \frac{\varphi_h}{a}\left(\frac{1+4\lambda^2}{c\pi} - 1\right) \qquad (8)$$

From equation 7, we obtain $\frac{\varphi_h}{a} \approx 0.53$ by inserting the experimental values of the ratio $\frac{E'_e}{E'_d}$ which is 1.33 (obtained from **Fig. 3f** and **5f**). $\frac{\varphi_h}{a} = 0.53$ can be used to calculate the values of *c* in equation 8 by inserting the experimental value obtained from $\frac{H'_e}{H'_d} = 1.5$ (obtained from **Fig. 3f** and **5f**). This gives $c \approx 4193$.

From this model, it can be seen how the spatial configuration of the crystals can drastically affect the values of the modulus and the hardness obtained experimentally with indentation. In particular, because of the presence of horizontal crystals, the modulus and hardness values for enamel will be always higher compared to those of dentine.

**Supplementary Discussion 9: Effect of solvent composition, environmental conditions, and substrate on the secondary structure conformation of the ELR matrix.**

The SEM results demonstrated similar ELR fibrillar structures when prepared using both DMF/DMSO (9/1 ration) (**Fig. 1a**) and ethanol/water (9/1 ration) (**Supplementary Fig. 43a**) solvent mixtures. Furthermore, ELR coatings prepared from both solutions exhibited similar secondary structure conformations (**Supplementary Fig. 3** and **Supplementary Fig. 43b**), leading to comparable mineralization outcomes including recreation of structure and restoration of properties (**Supplementary Fig. 43c – f** and **Fig. 3d – f**). Thus, motivated by these results, we further investigated the effect of different ratios of ethanol/water solvent mixture, drying speed, crosslinker concentration, and different substrates to deposit the ELR material on the secondary structure conformation of the ELR coatings. First, we explored the effect of varying the concentration of ethanol (0, 25, 50, and 85%) in the ethanol/water solvent mixture on the secondary structure conformation of ELR coating. The results demonstrated that increasing ethanol in the ethanol/water mixture increases the drying speed of the ELR coatings (**Supplementary Fig. 44a**) but without affecting their secondary structure conformation (**Supplementary Fig. 44b**). Furthermore, no differences were observed in the secondary structure conformation of the ELR coating when prepared using varying concentrations of glutaraldehyde crosslinker (0.1, 0.5, 1.0, and 1.5%) (**Supplementary Fig. 44c**). Also, we did not observe any differences in the secondary structure composition of the ELR coatings when prepared on top of different substrates including enamel and PDMS (**Supplementary Fig. 4**).

Overall, these results demonstrate the robustness of our technology in preserving the structural properties of ELR coatings independently of the type of solvent used, ethanol concentration, drying rate, crosslinker concentration, or substrate used. Furthermore, structural properties are essential for defining the resulting functional performance, and the ability to maintain these properties across various environmental conditions represents an important translational advantage of our technology.

**Supplementary Discussion 10: Confirmation of the strong interfacial bonding (i.e., integration) between the newly formed enamel and native enamel/dentine.**

Using TEM, we confirmed that the ELR matrix facilitates apatite crystal extension along the crystallographic c-axis **(Fig. 2a and Fig. 4)** mediated by an amorphous-to-crystalline transformation [9] during epitaxial remineralization, as previously reported[10]. High resolution TEM analysis exhibited an indistinguishable boundary between the newly grown mineral layer and the underlying native enamel, confirming a crystallographic integration between them **(Fig. 4)**. In addition, we have tested the mechanical properties and microtribological behaviour of the remineralized enamel compared to native and acid-etched enamel. The results revealed that the ELR matrix guided the remineralization of the acid-etched enamel, recreating the complex architecture **(Fig. 2d and g, Fig. 3d and e)**, restoring mechanical properties **(Fig. 3f)**, and regaining the CoF and wear properties **(Fig. 3g and h)** of healthy enamel. Together, these results indicate a strong interfacial integration between the remineralized layer and the native enamel tissue[10]. To further confirm this interfacial integration, we performed extensive tooth brushing (equivalent to ~1 year of brushing) and abrasion tests (equivalent to ~3.5 years of tooth attrition, chewing, and grinding). The results revealed that the remineralized enamel exhibited no significant drop in mechanical properties after brushing **(Fig. 6b)** and similar wear in terms of volume and height loss compared to healthy enamel during attrition tests **(Fig. 6f)**. All these results evidence a strong interfacial adhesion between the native enamel tissue and the remineralized layer.

**Supplementary Discussion 11: Summary of the key mechanistic features of the ELR mediated remineralization of enamel.**

1. $Ca^{2+}$, $PO_4^{3-}$, and $F^{1-}$ ions from the mineralization solution can diffuse into the ELR matrix to trigger the growth of organised (i.e., aligned) HAp nanocrystals even in the absence of underlying crystals, as we had previously reported[1].
2. Remineralization of the apatite nanocrystals embedded within the ELR matrix (**Fig. 2a**) and ELR coated enamel surface (**Fig. 4**) demonstrated epitaxial crystal growth preferentially along the c-axis (**Fig. 2a**) which may arise by the fusion and transformation of prenucleation clusters into crystalline structures along the c-axis, as previously reported[11,12]. This finding was supported by simulations, which revealed that more energy is required to remove ELR fragments bound on the a-axis than the c-axis of the apatite nanocrystal (**Supplementary Fig. 11**), as reported previously for a small 12-mer amelogenin sequence[13], thus energetically favouring epitaxial crystal growth along the c-axis.
3. The combination of the epitaxial mineralization (point 2) and the capacity to promote aligned nanocrystal growth (point 1), facilitate the recreation of the different anatomical structures of the native enamel upon remineralization.
4. Similarly, ELR coating deposited over exposed dentine also triggers epitaxial growth of HAp nanocrystals from the mineralized collagen fibrils (MCFs) via lattice continuity (**Fig. 5e, (i), (ii)**) and facilitates their organized growth from the dentine surface. A similar extension and organization of enamel crystals from the dentine MCFs has been observed in early amelogenesis[14,15].
5. When the thickness of the ELR coating on the surface of enamel is equal or less than 10 µm, the presence of underlying enamel nanocrystals reduces the energy barrier and facilitates heterogeneous nucleation preferentially at the interface between the ELR matrix and the c-axis of the nanocrystals. In this way, mineralization happens faster from the enamel nanocrystals compared to the formation of mineralized spherulitic structures within the matrix. When the thickness of the ELR coating on the surface of enamel is greater than 10 µm, similar mineralization takes place from the surface of the enamel nanocrystals but also nucleation points within the bulk of the ELR matrix can trigger the growth of spherulites.
6. After mineralization, the ELR matrix is degraded. Previous studies have reported on the biodegradability of similar ELR materials under physiological conditions[16-18].

## 2. Supplementary Figures

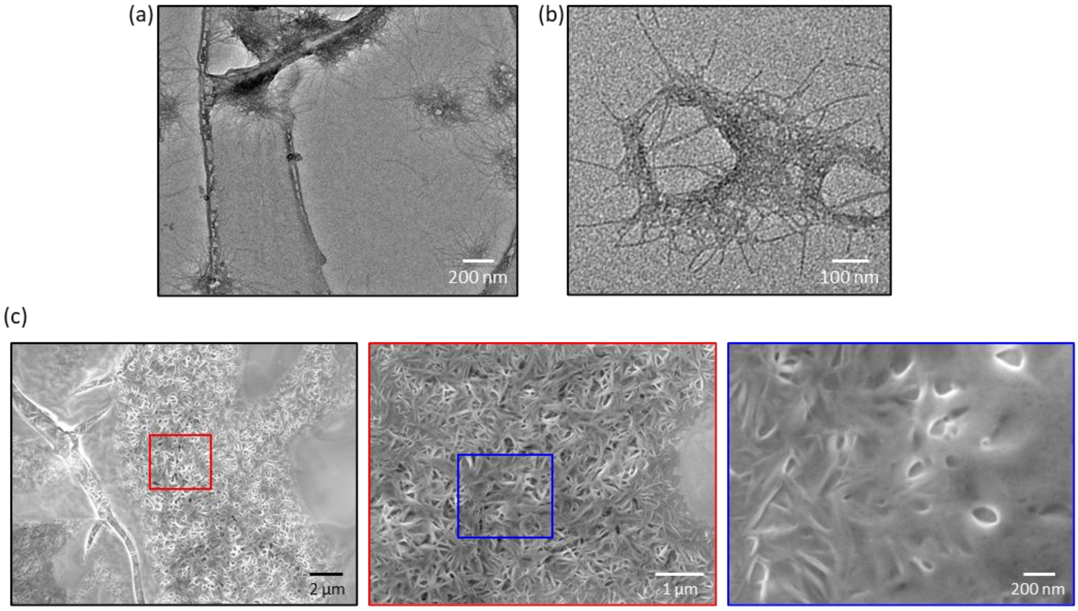


**Supplementary Fig. 1**. **TEM and SEM imaging of the ELR fibrils.**
**(a** and **b)** TEM images of the ELR fibrils at 1% w/v ELR concentration and 1.5 mM $Ca^{2+}$ ions without crosslinking, exhibiting fibrillar morphology. **(c)** SEM images showing aggregation of ELR fibrils when prepared using 5% w/v ELR solution containing 1.5 mM $Ca^{2+}$ ions without crosslinking. **(a, b)** was the confirmatory TEM experiment for main Fig. 1a from n = 5 independent experiments. For **(c)**, n = 3 independent experiments.

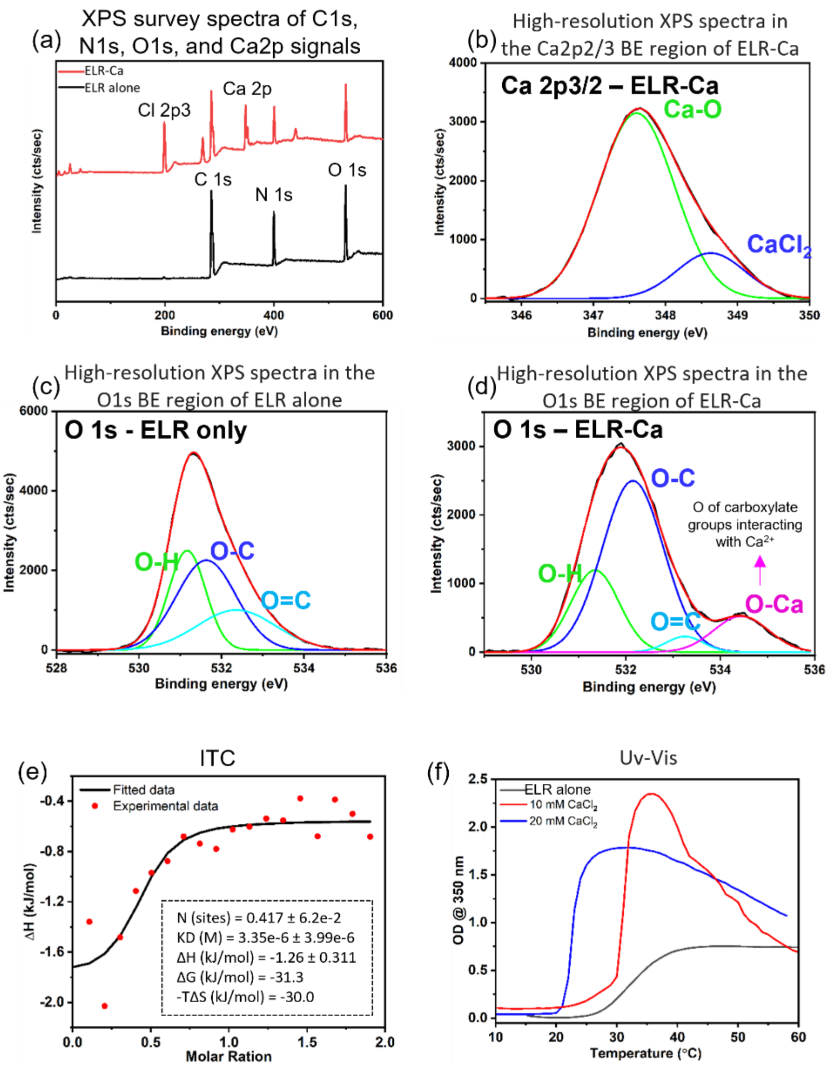


**Supplementary Fig. 2: Characterization of ELR-Ca interactions.**
**(a)** XPS survey spectra of C1s, N1s, O1s, and Ca2p signals. **(b)** High-resolution XPS spectra in the Ca2p2/3 BE region of ELR-Ca. **(c)** High-resolution XPS spectra in the O1s BE region of ELR alone. **(d)** High-resolution XPS spectra in the O1s BE region of ELR-Ca indicating interactions between $Ca^{2+}$ ions and carbonate group of ELR backbone. **(e)** Isothermal calorimetry (ITC) thermograph showing weak or non-specific interactions between 1.0 mM $Ca^{2+}$ ions and 100 µM ELR molecules. **(f)** UV-Vis spectra graph showing reduction in the transition temperature ($T_t$) as a result of Ca binding to the ELR molecules.

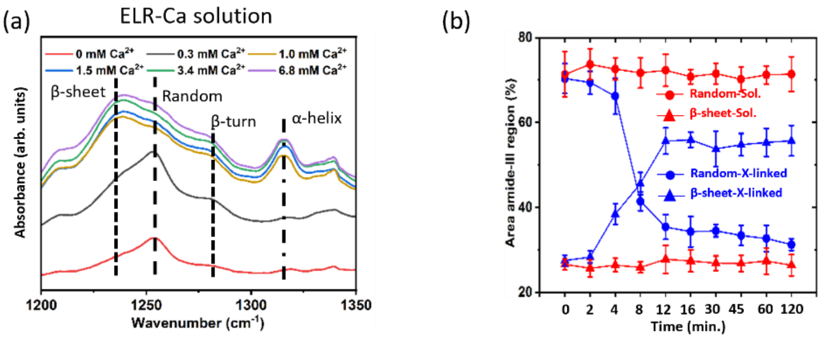


| $Ca^{2+}$ conc. | α-helix | β-turn | β-sheet | Random | Random:β-sheet | β-sheet + β-turn |
|---|---|---|---|---|---|---|
| **0.0 mM** | 6.2 | 5.2 | 46.0 | 43.4 | 0.95 | 51.2 |
| | 6.0 | 2.8 | 43.5 | 37.0 | 0.85 | 46.3 |
| | 6.4 | 7.6 | 48.5 | 49.8 | 1.02 | 56.1 |
| **AVG** | 6.2 | 5.2 | 46.0 | 43.3 | 0.94 | 51.2 |
| **SD** | 0.2 | 2.4 | 2.5 | 6.4 | 0.085 | 4.9 |
| | | | | | | |
| **1.5 mM** | 6.9 | 7.8 | 49.9 | 27.9 | 0.55 | 57.7 |
| | 6.8 | 10.3 | 53.1 | 30.0 | 0.56 | 63.4 |
| | 7.0 | 12.8 | 56.3 | 32.1 | 0.57 | 69.1 |
| **AVG** | 6.9 | 10.3 | 53.1 | 30.0 | 0.56 | 63.4 |
| **SD** | 0.1 | 2.5 | 3.2 | 2.1 | 0.01 | 5.7 |
| | | | | | | |
| **3.4 mM** | 8.1 | 21.9 | 55.1 | 16.8 | 0.30 | 77.0 |
| | 7.8 | 19.0 | 52.7 | 15.2 | 0.29 | 71.7 |
| | 8.4 | 23.2 | 57.5 | 18.4 | 0.32 | 80.7 |
| **AVG** | 8.1 | 21.4 | 55.1 | 16.8 | 0.30 | 76.46 |
| **SD** | 0.3 | 2.15 | 2.4 | 1.6 | 0.01 | 4.52 |
| | | | | | | |
| **6.8 mM** | 7.5 | 24.2 | 56.4 | 14.4 | 0.25 | 80.6 |
| | 5.3 | 23.0 | 54.1 | 13.3 | 0.24 | 77.1 |
| | 7.9 | 25.4 | 58.7 | 15.6 | 0.26 | 84.1 |
| **AVG** | 6.9 | 24.2 | 56.4 | 14.4 | 0.25 | 80.6 |
| **SD** | 1.4 | 1.2 | 2.3 | 1.15 | 0.01 | 3.5 |

**Supplementary Fig. 3: Secondary structure conformation of ELR in solution and in matrix at different Ca concentration.**
**(a)** FTIR spectra showing amide–III region (1200-1350 $cm^{-1}$) of ELR-Ca solution at different $Ca^{2+}$ concentration. **(b)** Evolution of the % area of amide-III region as a result of subsequent drying and crosslinking of ELR-Ca solution containing 1.5 mM $Ca^{2+}$ ions. **(c)** Secondary structure composition of dried and cross-linked ELR membranes prepared at different $Ca^{2+}$ ion concentrations. The values shown are percentage amide-III region.

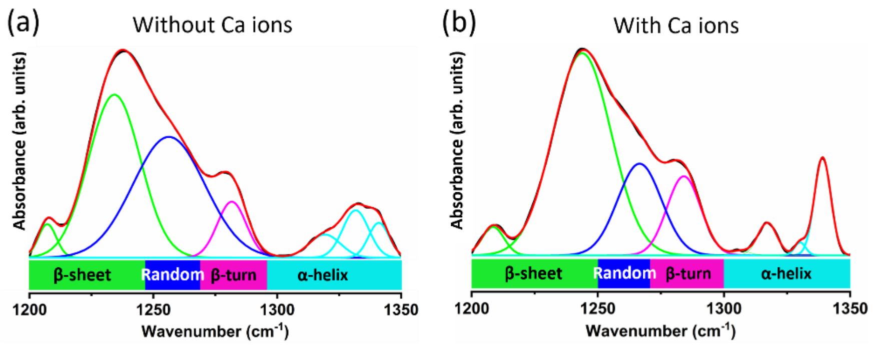


(c) Secondary structure composition of ELR coatings on enamel

| | β-sheet (%) | Random (%) | β-turn (%) | α-helix (%) | Random/β-sheet | β-sheet + β-turn (%) |
|---|---|---|---|---|---|---|
| **Without Ca ions** | 41.08 | 39.45 | 7.33 | 12.12 | 0.96 | 48.41 |
| **With Ca ions** | 53.96 | 27.39 | 7.79 | 10.85 | 0.50 | 61.75 |

**Supplementary Fig. 4. Secondary structure composition of ELR coatings on enamel surface.** FTIR spectra showing deconvoluted amide–III region (1200-1350 $cm^{-1}$) recorded on ELR coatings prepared on enamel surface **(a)** without and **(b)** with 1.5 mM $Ca^{2+}$ ions. **(c)** Table showing % of ordered (β-sheet + β-turn) and disordered (random) conformation.

**Discussion:** We investigated the role of both the free $Ca^{2+}$ ions present in the solution and the $Ca^{2+}$ ions present in the enamel nanocrystals on the self-assembly of the ELR molecules during coating. To do this, we deposited an ELR coating without and with 1.5 mM $Ca^{2+}$ ions on the enamel surface and analysed the secondary structure conformation of the coated ELR using FTIR. Additionally, we also investigated the conformation of the ELR coating without $Ca^{2+}$ ions on PDMS, as a non-enamel substrate. We observed that the percentage of ordered structures (i.e., β-sheet + β-turn) in the ELR coatings without $Ca^{2+}$ ions on top of enamel (48.41%) was lower compared to ELR coatings with $Ca^{2+}$ ions on enamel (61.75%) (**Supplementary Fig. 4**) but was comparable to the ELR coating without $Ca^{2+}$ ions on PDMS substrates (51.2%) (**Supplementary Fig. 3c**). These results indicate that free $Ca^{2+}$ ions from the solution interact with the ELR molecules to form inter-molecular ionic bridges between ELR molecules via electrostatic interactions that leads to ELR self-assembly. These results are consistent with our computational modelling results (**Fig. 1d (i), (ii) and Supplementary. Fig. 9**) and in agreement with previous reports[19]. In contrast, $Ca^{2+}$ ions present in enamel crystals are in a CaP phase and lack the ability to form inter-molecular ionic bridges between ELR molecules, resulting in less ordered structures in the ELR coatings prepared without $Ca^{2+}$ ions. Thus, these results demonstrate that the presence of free $Ca^{2+}$ ions in the solution is crucial for the ELR self-assembly during coating on the enamel surface.

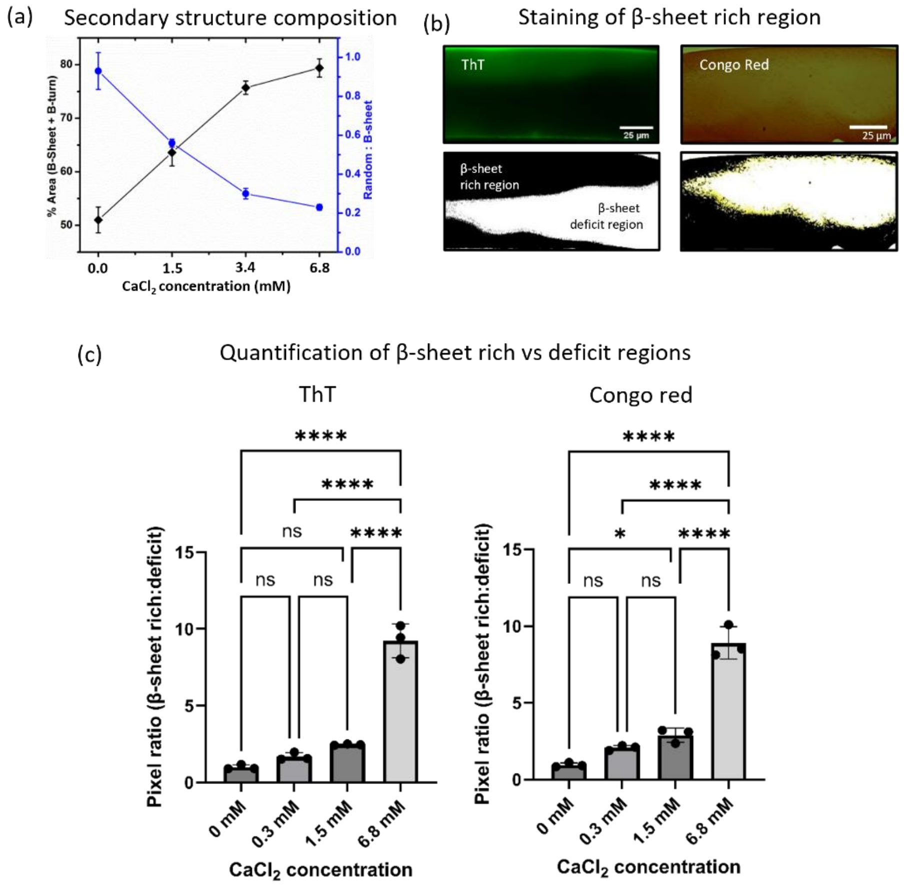


**Supplementary Fig. 5: Quantification of secondary structure conformation and staining of β-sheet rich regions in crosslinked ELR membranes.**

**(a)** Graph showing different levels for various secondary structure conformations (i.e., β-turn, β-sheet, and random) and their ratio as a function of different concentrations of $Ca^{2+}$ ions present in dried and crosslinked ELR membranes. Analysis was performed by deconvoluting amide-III (1200-1350 cm-1) region in the FTIR spectra. **(b)** An example showing ThT and Congo Red staining of the cross-section of the membrane containing 0 mM $CaCl_2$. **(c)** Histologically stained cross-sections of ELR membranes containing different concentration of $Ca^{2+}$ ions. Post staining, number of pixels from stained (β-sheet rich) and non-stained (β-sheet deficit) regions were calculated using ImageJ software for computing their ratio (n = 3 samples). Data are presented as mean ± SD. Statistical significance was analysed using two-sided one-way ANOVA (Tukey test) in GraphPad Prism ver. 10. In **(c)** **** represents significant difference $p < 0.0001$, * represents $p = 0.0151$, and ‘ns’ represents no significant difference.

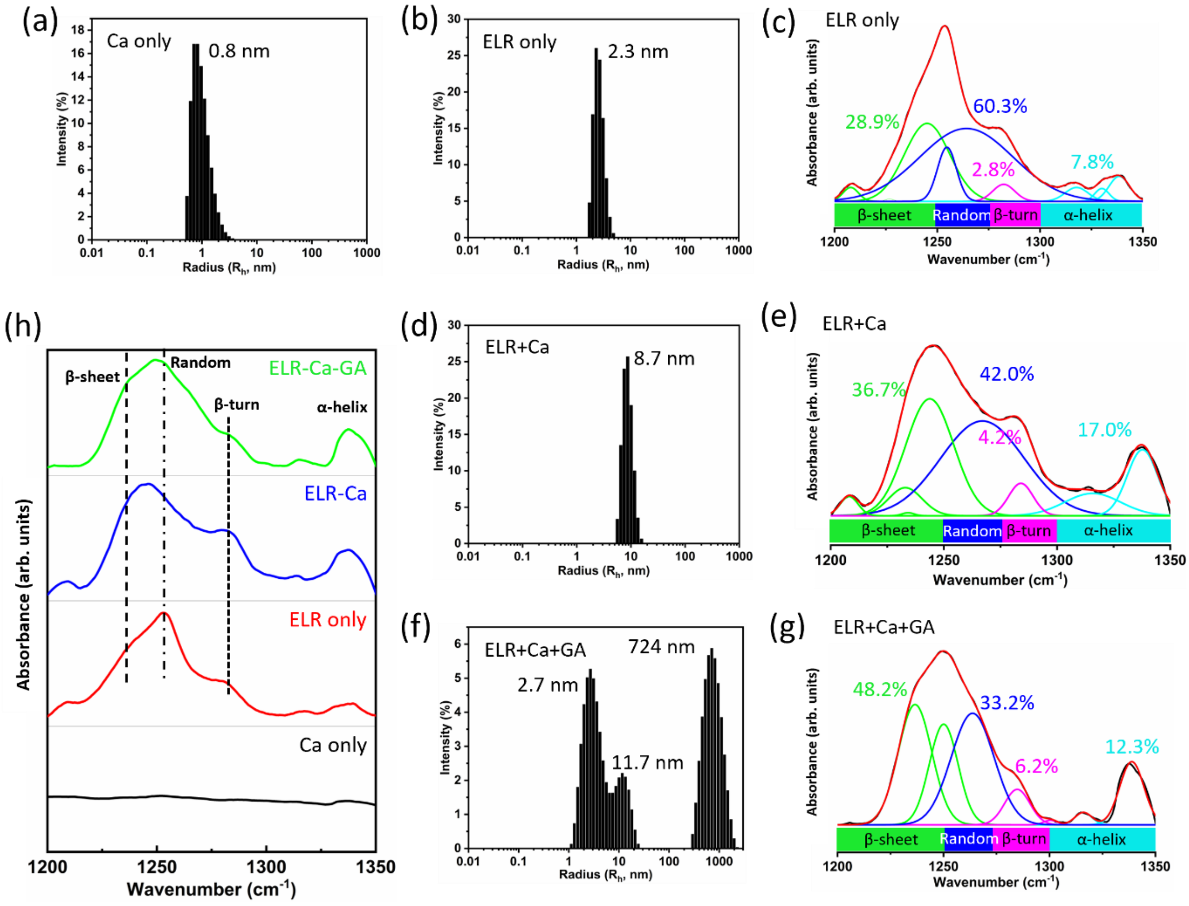


**Supplementary Fig. 6: Ca ions and crosslinking regulate the formation of supramolecular ELR structures in solution.**

Dynamic light scattering (DLS) and FTIR analyses of solutions prepared using ethanol/water (9/1) solvent mixture and containing **(a)** 1.5 mM Ca ions only, **(b, c)** 5% (w/v) ELR only, **(d, e)** ELR + Ca, and **(f, g)** ELR + Ca + Glutaraldehyde (GA). **(h)** FTIR spectra showing amide-III (1200-1350 cm$^{-1}$) regions from above solutions.

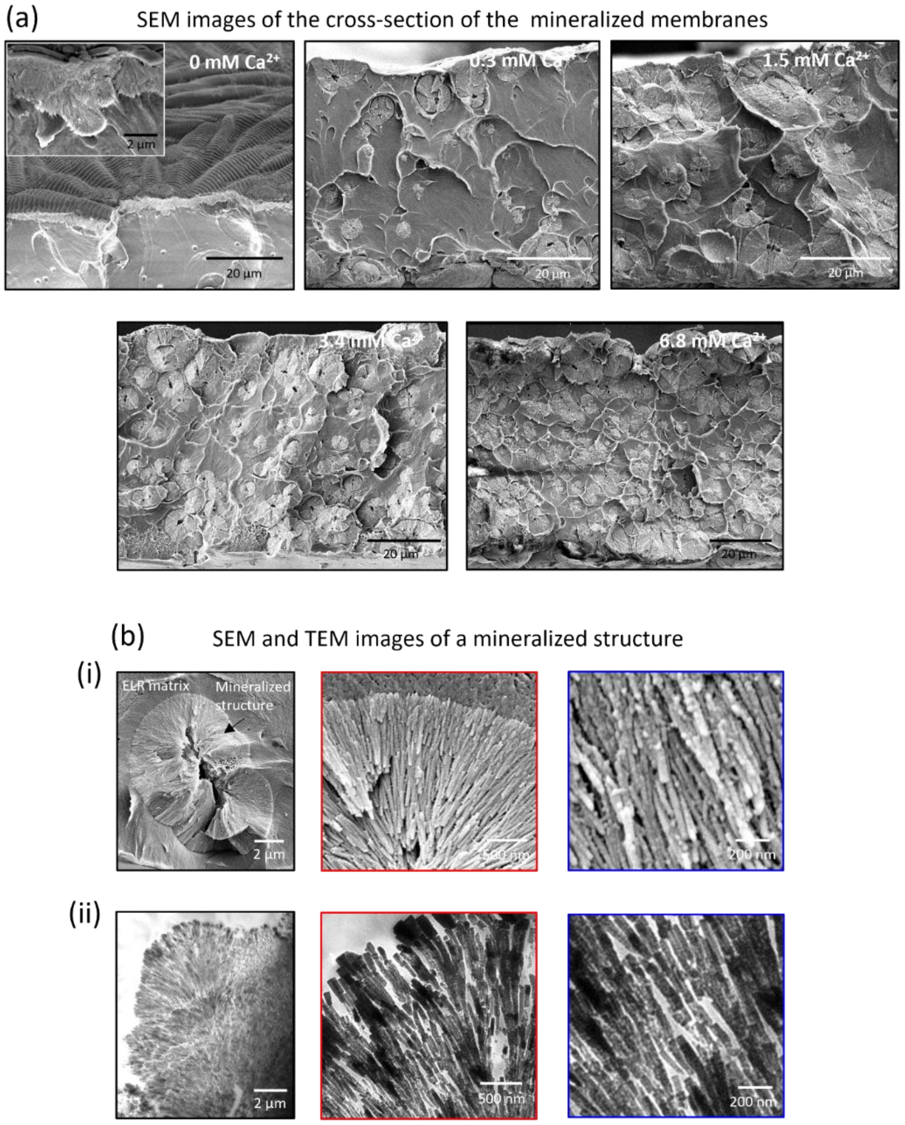


**Supplementary Fig. 7: Increasing the Ca concentration in ELR matrix increases mineralization.** SEM images showing the cross-section of the ELR membranes mineralized for 10 days. Increasing the Ca concentration (from 0 to 6.8 mM Ca$^{2+}$) during ELR membrane fabrication resulted in the increased number of nucleation points for mineralization. **(b-i)** SEM and **(b-ii)** TEM images (low to high magnification) of a mineralized structure grown within the ELR membrane containing 1.5 mM Ca$^{2+}$. For **(a, b)**, representative images from n = 3 independent experiments are used.

**Description:**

In the presence of an inorganic template, ELR matrix triggers epitaxial growth within the matrix, as presented in two different ways. First, by embedding HAP nanocrystals within the ELR matrix as in **Fig. 2a** and then by presenting enamel nanocrystals to the ELR matrix. In both cases, the nanocrystals serve as an inorganic template where new nanocrystals template from and grow epitaxially. We have demonstrated this experimentally (**Fig. 2a, c, d, Fig. 3d, e, and Fig. 4a, b, e, h**) and supported the data with simulations (**Supplementary Fig. 11**, **Supplementary Discussion 11**).

However, in the absence of an inorganic template, the ELR matrix promotes the growth of oriented nanocrystals (**Supplementary Fig. 7a**) but organised as spheres, as reported previously[1].

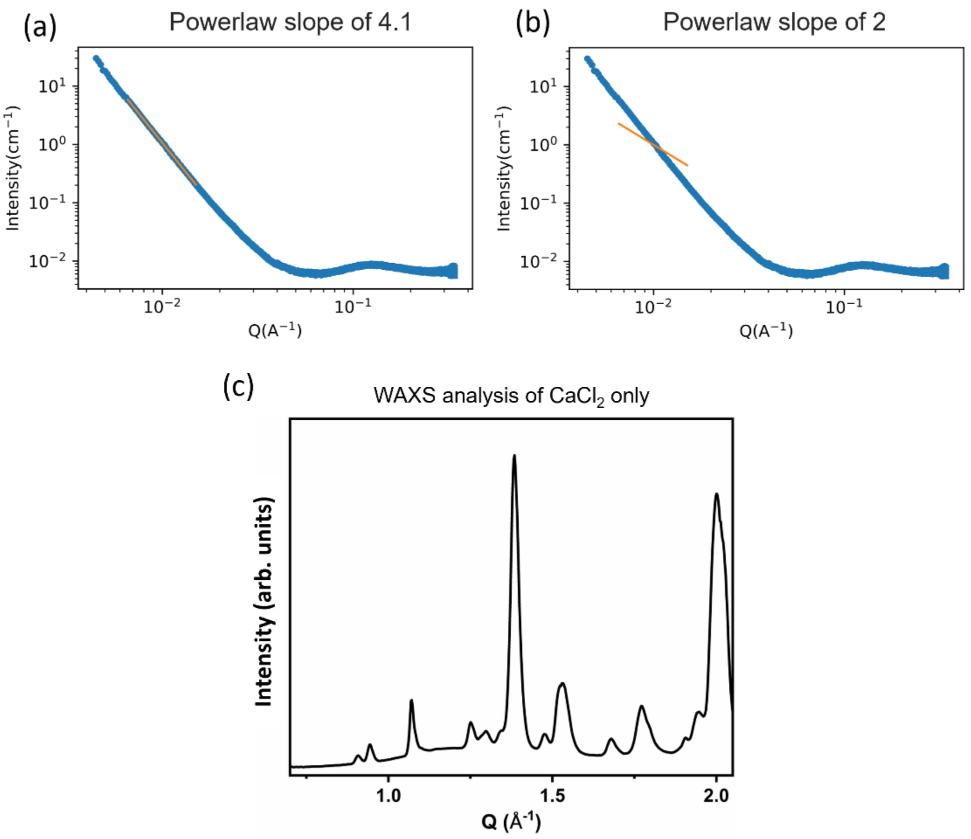


**Supplementary Fig. 8: X-ray scattering analysis.**
SAXS data of ELR fibrils showing a small broad peak at Q = 0.129 Å$^{-1}$ that corresponds to a d-spacing of 48.7 Å and is attributed to the width of an individual filament. Fitting of the SAXS data using SASview software with **(a)** the initial powerlaw slope of 4.1 and **(b)** powerlaw slope of 2. **(c)** WAXS analysis of $CaCl_2$ crystals.

**Description:**
Using q values from 1D WAXS, we calculated the d-spacings and 2θ angles for $CaCl_2.2H_2O$ using the following expressions:

$d = 2\pi/q$ (Eq. 1)

$q = 4\pi \sin\theta/\lambda$ (Eq. 2)

Cu Kα radiation exhibiting a wavelength (λ) of 1.5418 Å was used to compute above 2θ values. Powder Diffraction File (PDF) 01-075-0305 from the International Centre for Diffraction Data (ICDD) database was used to calculate the Miller indices as shown below.

Table showing q values, d-spacing, 2θ (degrees), and Miller indices for $CaCl_2.2H_2O$ crystals.

| **q (Å$^{-1}$)** | **d-spacing (Å)** | **2θ (degrees)** | **Miller indices** |
|---|---|---|---|
| 1.07 | 5.87 | 15.1 | (001) plane |
| 1.25 | 5.03 | 17.6 | (002) plane |
| 1.38 | 4.52 | 19.5 | (110) plane |
| 1.53 | 4.11 | 21.6 | (111) plane |
| 1.77 | 3.55 | 25.1 | (112) plane |
| 2.00 | 3.14 | 28.4 | (211) plane |

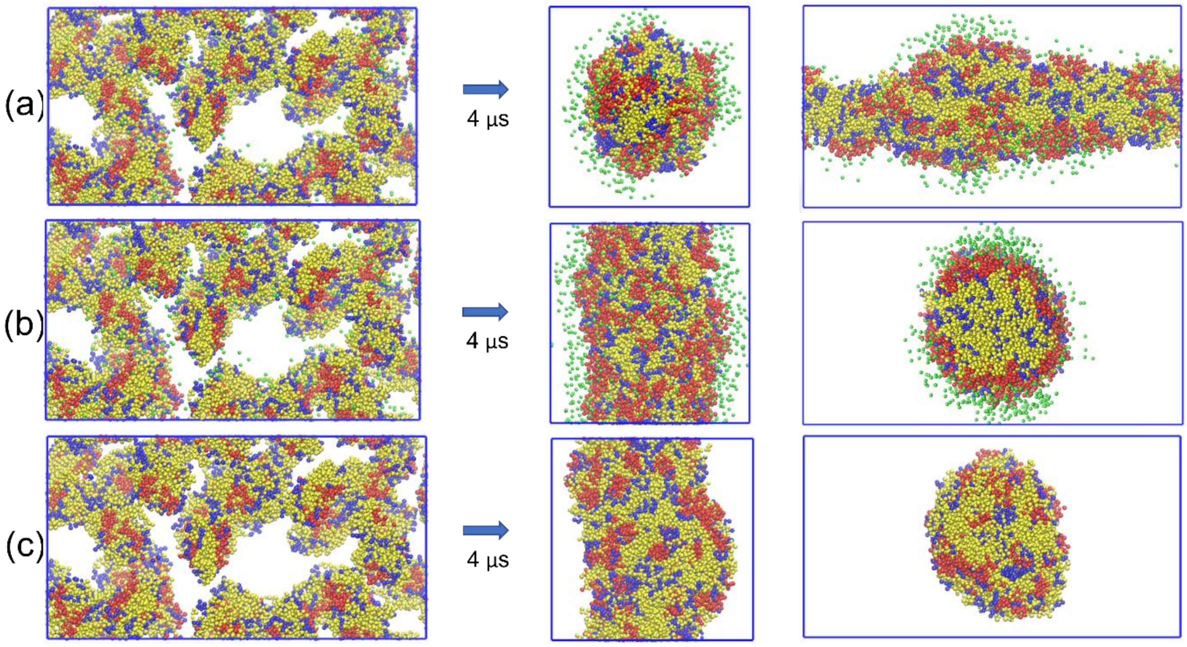


**Supplementary Fig. 9: Coarse-grained simulation confirms ELR filament formation in presence of $Ca^{2+}$ ions and their assembly into fibril.**

Thirty equilibrated protein molecules were inserted into a 16x16x30 nm aqueous rectangular cuboid box and equilibrated with **(a)** coiled secondary structure of the statherin motif and beta-sheet secondary structure with a 0.2 M concentration of $Ca^{2+}$ ions, **(b)** coiled secondary structure for the entire protein with a 0.2 M concentration of $Ca^{2+}$ ions, and **(c)** coiled secondary structure of the statherin motif and beta-sheet secondary structure without $Ca^{2+}$ ions. The statherin motif is represented by red beads, $Ca^{2+}$ ions in green, VPGKG by blue, and all others by yellow beads. In the case of (a) and (b) statherin motifs are orientated on the surface of the aggregate where $Ca^{2+}$ ions are present, however only the combination of the presence of $Ca^{2+}$ ions and the secondary structure of (a) promote formation of elongated filaments.

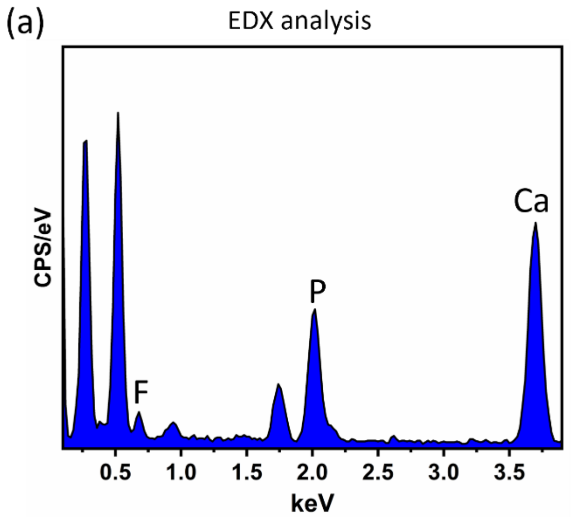


(b) Elemental composition

| Element | Atomic % |
|---|---|
| Ca | 8.1 |
| P | 5.05 |
| F | 1.49 |

Ca/P = 1.60
P/F = 3.38

**Supplementary Fig. 10: Chemical analysis of mineralized ELR fibers.**
(a) EDX analysis confirms the presence of flouride in the apatite nanocrystals. (b) The Ca/P (1.60) and P/F (3.38) ratios of the grown nanocrystals exhibited similar stoichiometric ration compared to flourapatite nanocrystals (Ca/P = 1.67 and P/F = 3), confirming the formation of flourapatite nanocrystals.

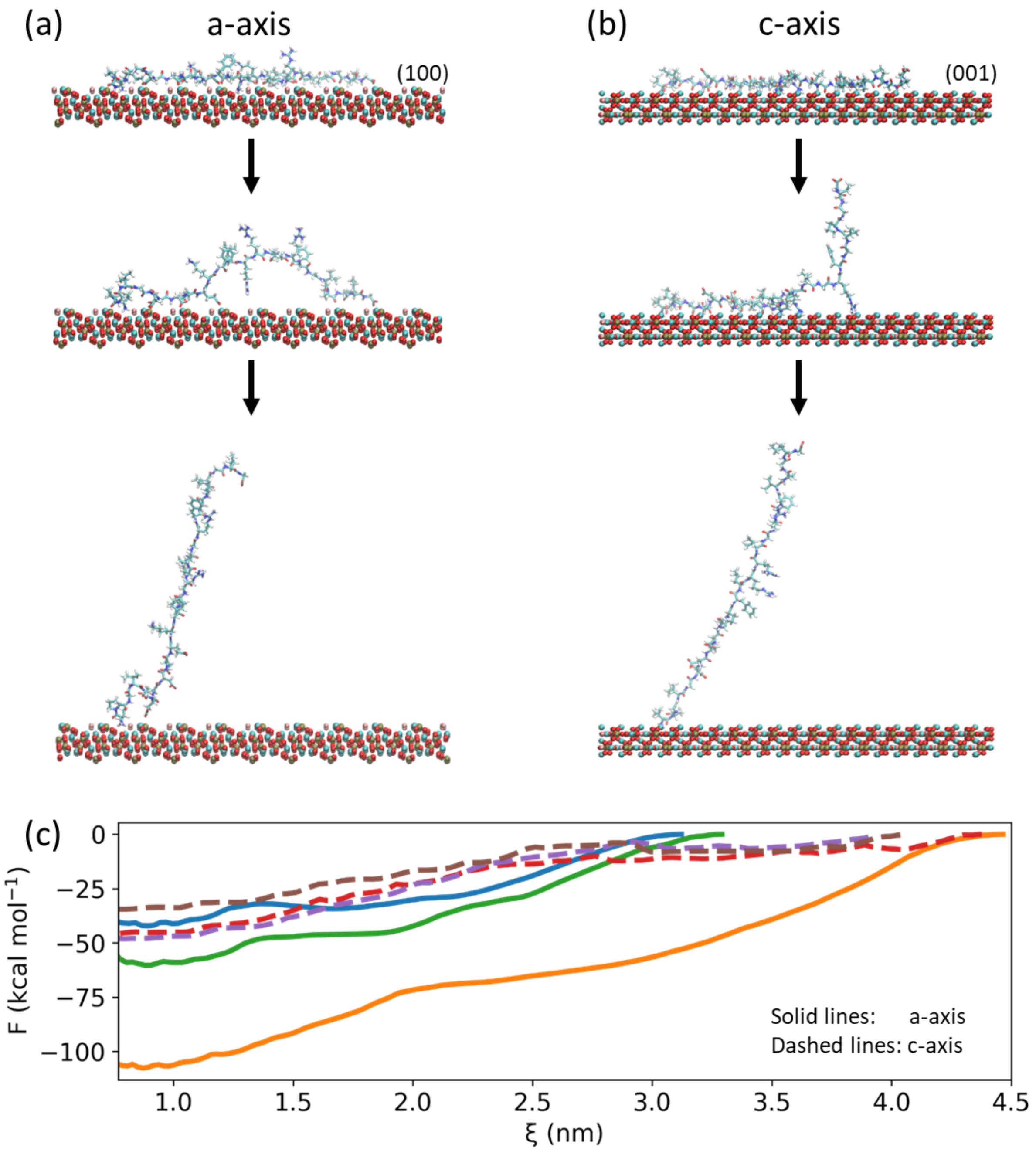


**Supplementary Fig. 11: Coarse-grained simulation showing detachment of a small ELR fragment from a- and c-axes of the flourapatite crystal.**

**(a,b)** A reduced representation of the statherin protein (VPGIGDDDEEKFLRRIGRFGVPGIG) was simulated in the CHARMM36[20] forcefield being pulled from the a fluorapatite crystal surface (along the z-axis normal to the a- and c-axes) in CHARMM-modified TIP3P water (not shown). **(c)** The potential of mean force was calculated by the WHAM[21] method finding that the peptide binds more favourably to the a-axis (solid line, 70.1 ± 32.76 kcal $mol^{-1}$) than the c-axis (dashed, 42.8 ± 6.81 kcal $mol^{-1}$). This result indicates that more energy is required to remove a ELR fragment from the a-axis than the c-axis and thus promotes growth of the crystal along the c-axis. The difference is expected to be significantly larger for an entire protein and larger so for a fibral of proteins. **(a, b):** In crystal structure, pink balls represents F, cyan represents Ca, red represents O, and golden brown represents P. **(c):** 3 solid and dashed lines represent 3 runs of each pulling simulation on a-axis and c-axis, respectively.

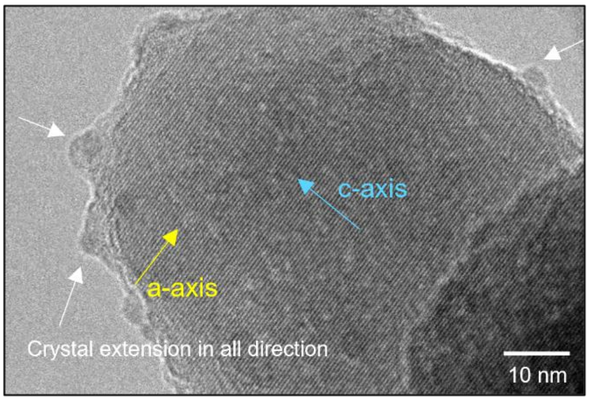


**Supplementary Fig. 12: Uncontrolled remineralization of synthetic HAp crystal in the absence of ELR matrix.**

TEM images showing irregular mineral growth along all the axis of the nanocrystal when mineralized in the absence of ELR matrix (n = 3 independent experiments).

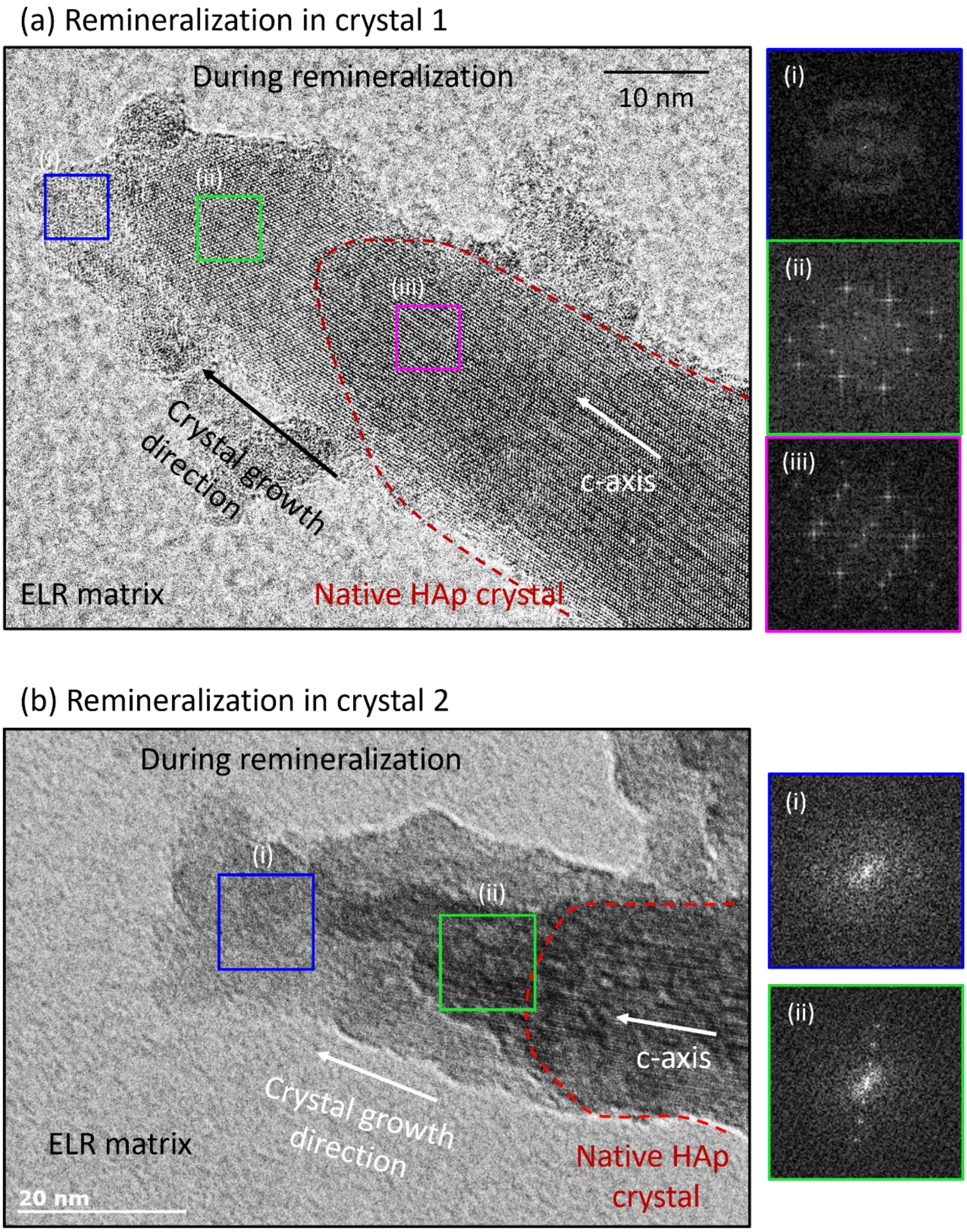


**Supplementary Fig. 13: TEM image showing crystal extension in the presence of ELR matrix.** **(a, b)** TEM images showing preferential crystal growth along the c-axis in the presence of ELR matrix in two different hydroxyapatite nanocrystals. Insets in both images show FFT graphs confirming amorphous (blue square) and crystalline (green square) regions. For **(a, b)**, representative images from n = 3 independent experiments are used.

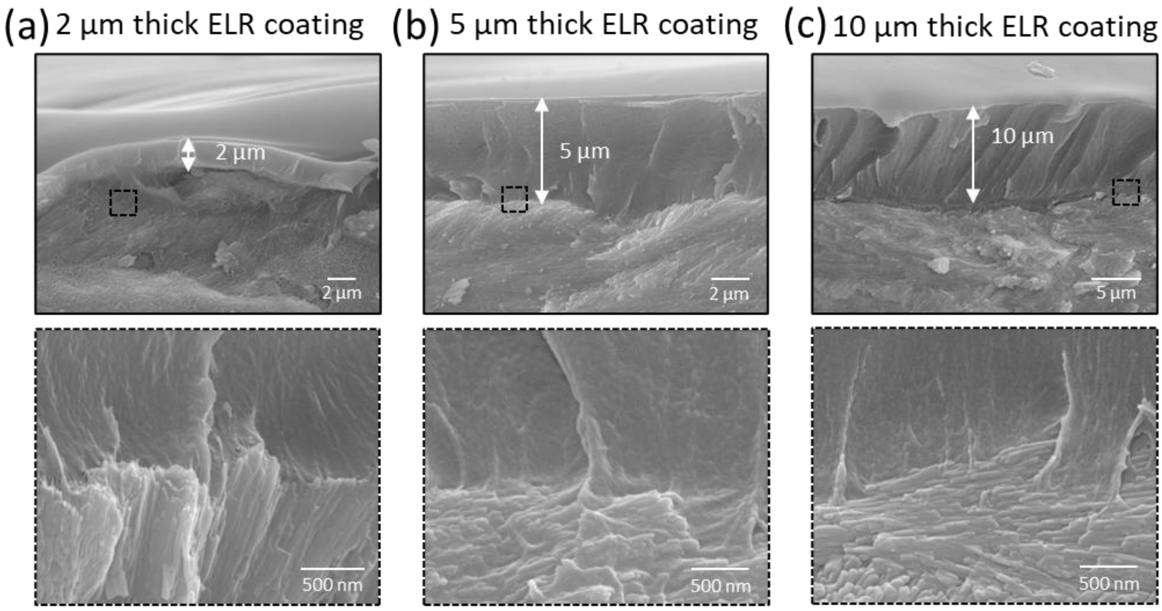


**Supplementary Fig. 14. Optimization of ELR coating thicknesses on enamel surface.**
SEM images showing ELR coating of **(a)** 2 µm, **(b)** 5 µm, and **(c)** 10 µm thickness on enamel surface. For **(a, b, c)**, representative images from n = 3 independent experiments are used.

**Description:** We conducted systematic experimentation to demonstrate the control over ELR coating thickness. We created 2 mm X 4 mm windows of enamel sections and drop casted different volumes of ELR solution (5% w/v ELR, 1.5 mM $Ca^{2+}$ ions, and 0.56% HDI) varying from 2 µL, 5 µL, and 10 µL (5 µL x 2 applications to prevent solution overflow).

**Rationale for using SEM imaging:** We used SEM observations to measure coating thicknesses throughout our study because it provides a high-resolution measuring method that we can use to assess coatings on different tooth areas including intricate anatomical regions. This level of precision is necessary to establish a precise coating protocol capable of predicting ELR coating thicknesses. Furthermore, our group has extensive experience in sample preparation and analysis using SEM to characterize ELR-based materials with nanoscale resolution[22-24].

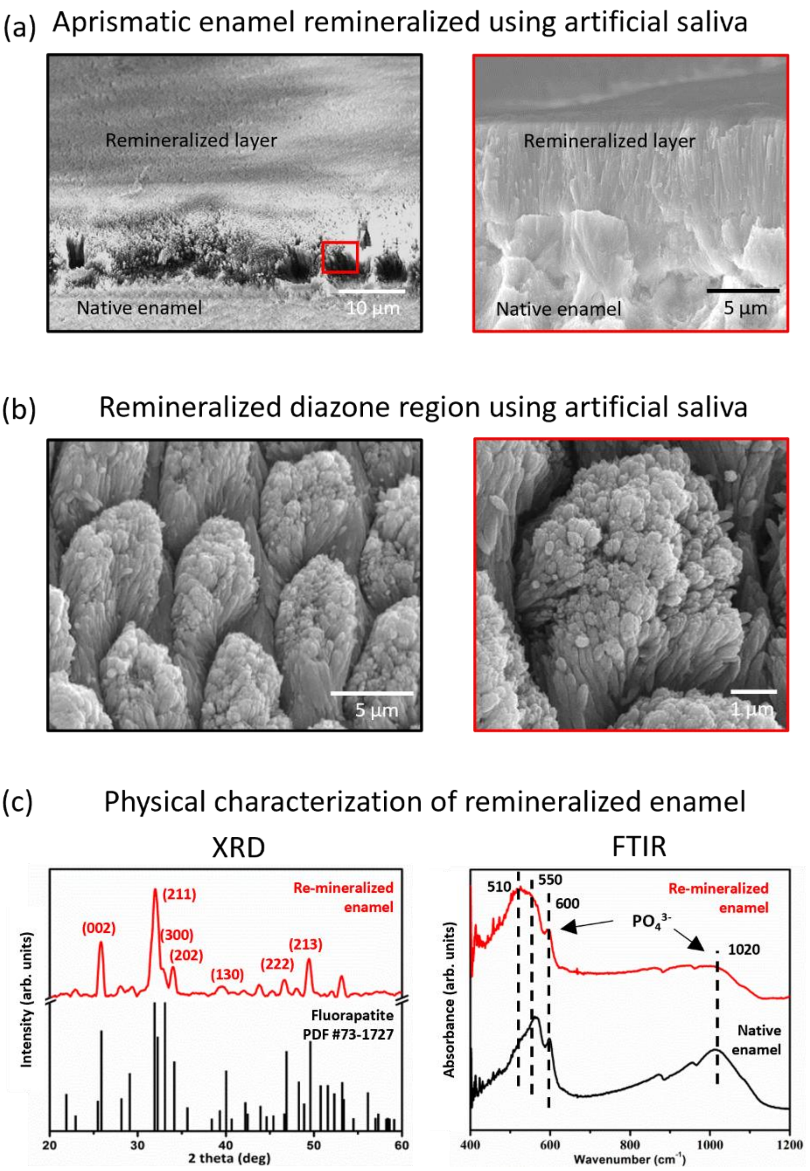


**Supplementary Fig. 15: Enamel remineralization using artificial saliva.**
SEM images confirming the capacity of our ELR matrix to remineralize different anatomical regions of enamel using artificial saliva. **(a)** mineralized layer on aprismatic enamel and **(b)** remineralized diazone region after 10 days of mineralization. **(c)** Physical characterization of remineralized enamel using XRD and FTIR analysis confirmed apatite growth. For **(a)** and **(b),** representative images from n = 3 and n = 7 independent experiments are used, respectively.

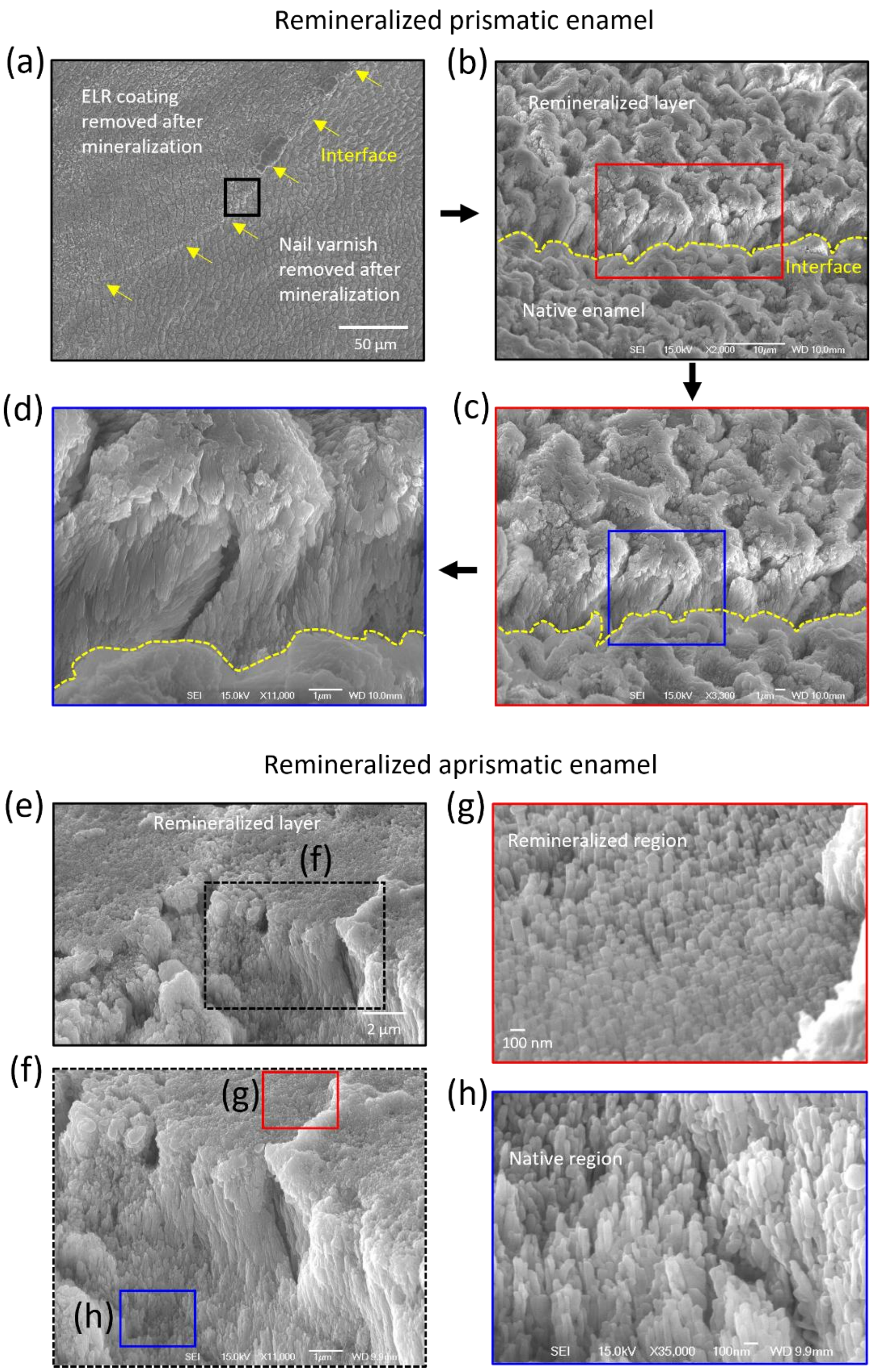


**Supplementary Fig. 16. Fabrication of mineralized layer on prismatic and aprismatic enamel regions.** SEM images showing uniform ~5 µm thick mineralized layer **(a, b, c, d)** on prismatic and **(e, f, g, h)** aprismatic enamel regions, recreating the microstructure of both anatomies. We used a nail varnish approach where half of the enamel surface was covered with nail varnish and the remaining half was coated with 5 µm thick ELR coating. Post mineralization, ELR coating and nail varnish were removed to expose both mineralized and native regions of enamel, respectively. For **(a - h)**, representative images from n = 3 samples are used.

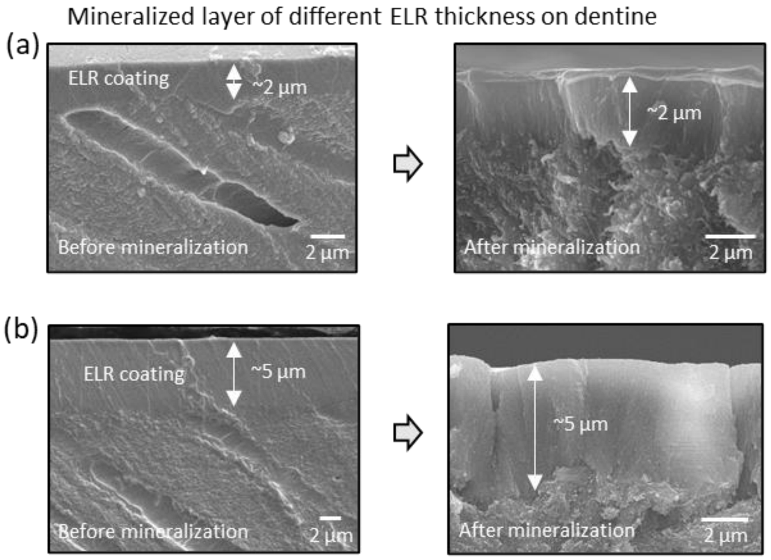


**Supplementary Fig. 17: Tuneability of mineral layer thickness on dentine.**
SEM images showing thickness of mineralized layers limited by the thickness of the ELR coatings on **(a and b)** dentine after 10 days of mineralization. These results confirm that the nanocrystal growth was limited by the thickness of the ELR matrix, generating a mineralized layer of equal thickness to the ELR coating. For **(a, b)**, representative images from n = 3 independent experiments are used.

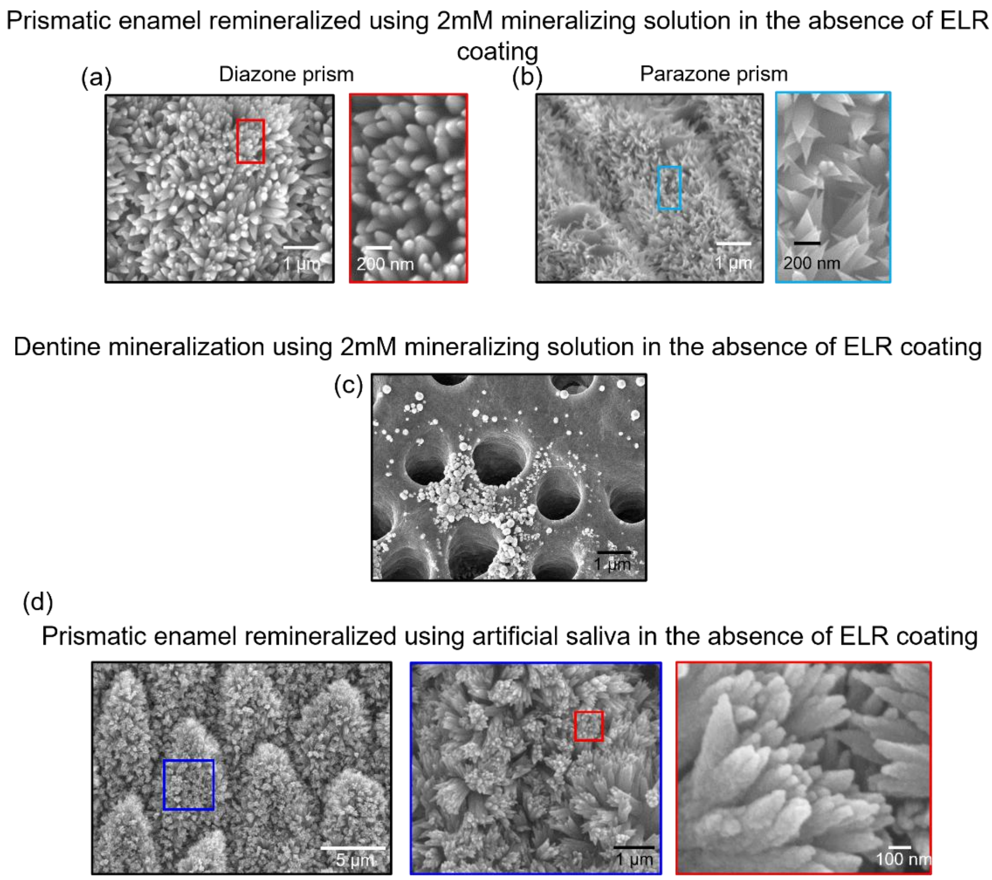


**Supplementary Fig. 18: Uncontrolled mineralization on enamel and dentine surface in the absence of ELR matrix.**
SEM images showing misoriented crystals grown on **(a)** diazone and **(b)** parazone regions of enamel and **(c)** dentine surface remineralized for 10 days in the absence of ELR coating. **(d)** Similarly prismatic enamel remineralized using artificial saliva too exhibited misoriented crystal growth in the absence of ELR coating. These results confirm the evident role of ELR matrix in guiding the epitaxial and oriented growth of apatite nanocrystals. For **(a, b, c)**, representative images from n = 3 independent experiments are used. For **(d)**, representative images from n = 4 independent experiments are used.

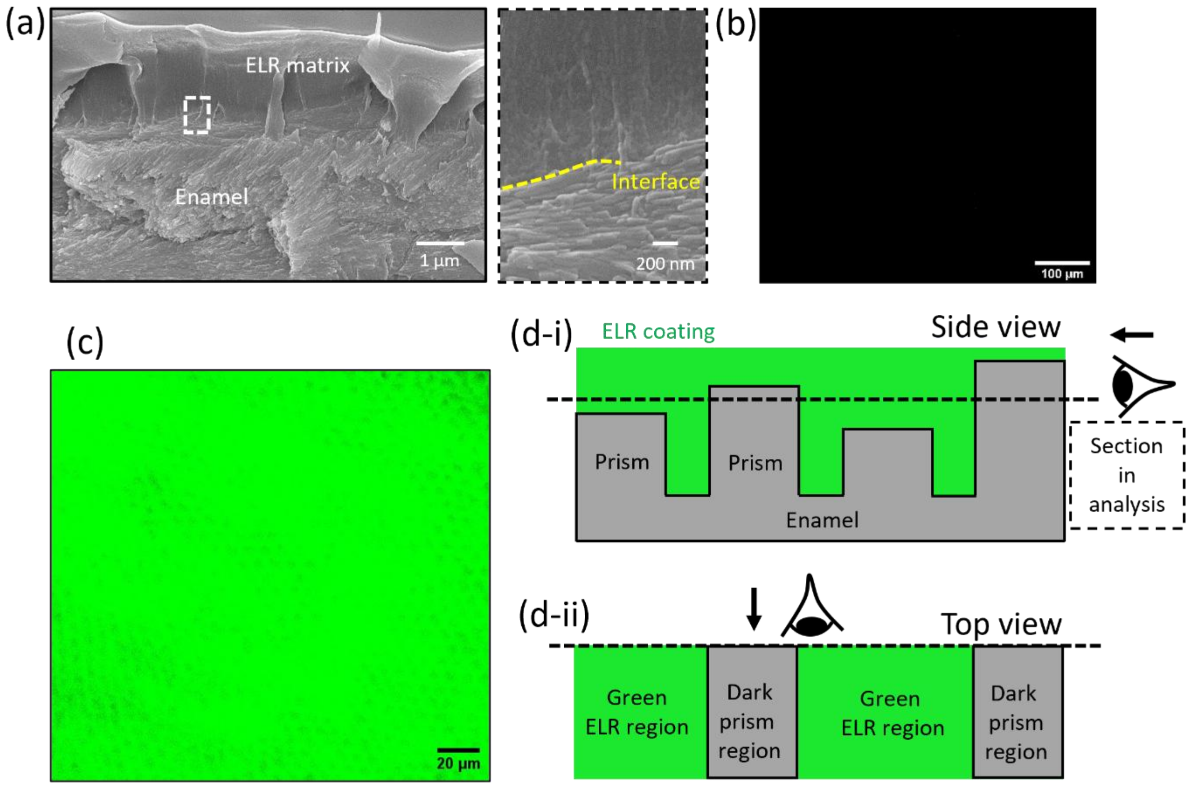


**Supplementary Fig. 19: SEM and confocal imaging.**
**(a)** SEM image showing 2 µm thick ELR coating on enamel surface and **(b)** confocal laser scanning microscopy (CLSM) image of bare enamel section (i.e., without ELR coating) after ThT staining. **(c)** CLSM image showing ThT staining of an ELR coated enamel surface. **(d)** Illustrations showing how different regions of the sample may appear more or less green, depending on the section of the image. The discontinuous or dark spot features observed in the image in panel (c) result from the sectioning of the confocal image. The ELR coating conforms closely to the underlying enamel topography and so even though the ELR coating is uniform, dark regions may appear depending on the depth at which the confocal image is taken. In other words, dark regions will appear when the focal plane of the confocal image goes through densely packed enamel crystals where the ELR matrix is unable to penetrate as shown in **d-i** and **d-ii**. In contrast, regions where the ELR is present appear bright green. For **(a - c)**, representative images from n = 3 independent experiments are used.

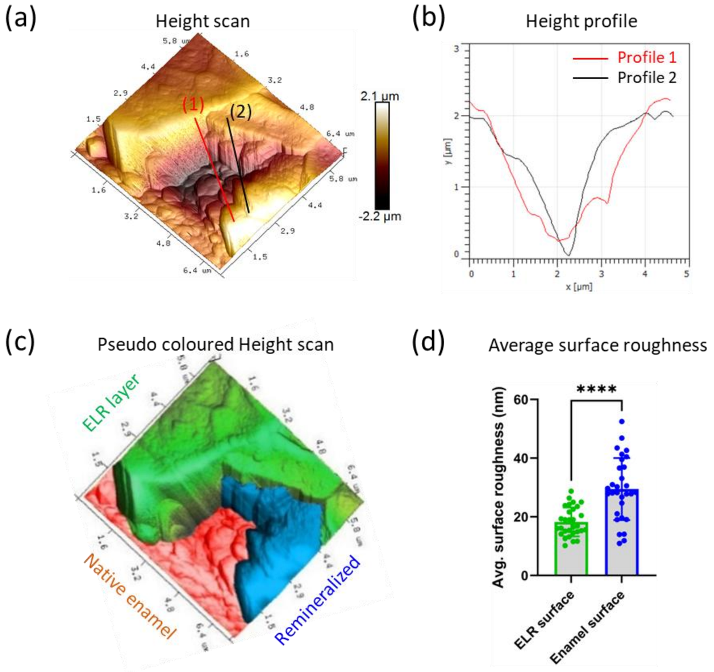


**Supplementary Fig. 20: Atomic force microscopic (AFM) imaging of mineralized enamel section.** **(a)** Original height scan of partially remineralized enamel surface. **(b)** Height profile generated from (a) using Gwyddion software showing 2 µm thick mineralized layer. **(c)** Pseudo coloured height scan showing the distribution of the ELR coating, mineralized layer and native region of enamel. **(d)** Estimation of the average surface roughness ($R_a$) from (c) showing significant difference between remineralized layer and ELR coating. Statistical significance was analysed using two-tailed Student's t-test using GraphPad Prism ver. 10. In **(d)** **** represents $p < 0.0001$.

**Description:** Using Gwyddion software, we generated a height profile that distinctly shows the height difference between the ELR coating and the remineralized regions (**Supplementary Fig. 20a, b**). In addition, we also calculated the average surface roughness ($R_a$) to differentiate between that of the ELR coating and of the mineralized layer. The ELR coating (marked with green) is smoother and therefore exhibited a lower $R_a$ compared to the rougher remineralized enamel (marked with blue) (**Supplementary Fig. 20d**).

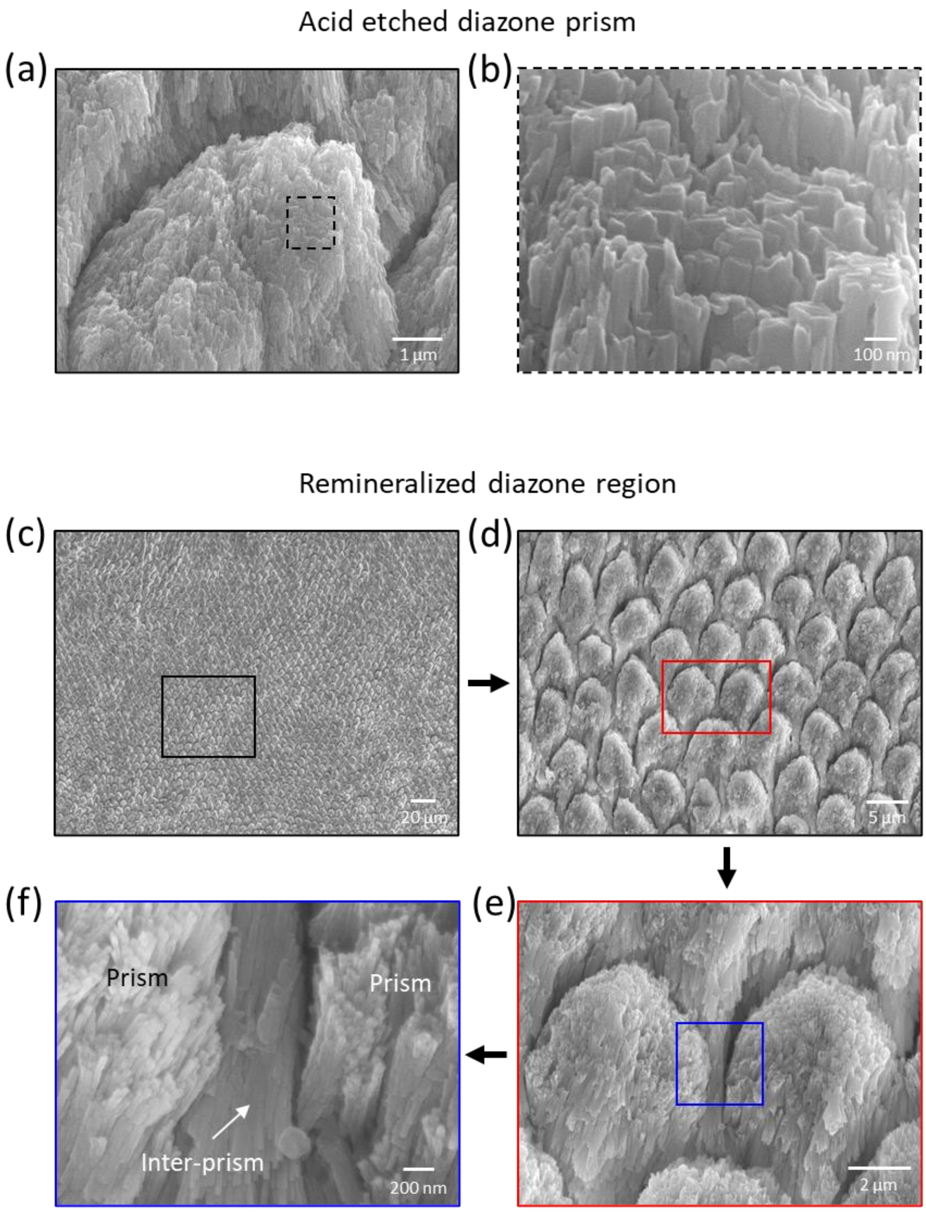


**Supplementary Fig. 21. Acid etched and remineralized diazone region of enamel.**
**(a)** SEM image showing acid etched diazone prism (37% phosphoric acid, 30 seconds). **(b)** High magnification SEM image showing the nanocrystal morphology and confirming that these nanocrystals remain oriented in the same direction as they were prior to acid treatment. **(c, d, e, f)** Low to high magnification SEM images showing remineralized diazone regions of enamel. These results demonstrate the uniformity of the mineralized layer over large and uneven surfaces of enamel. For **(a - e)**, representative images from n = 8 independent experiments are used.

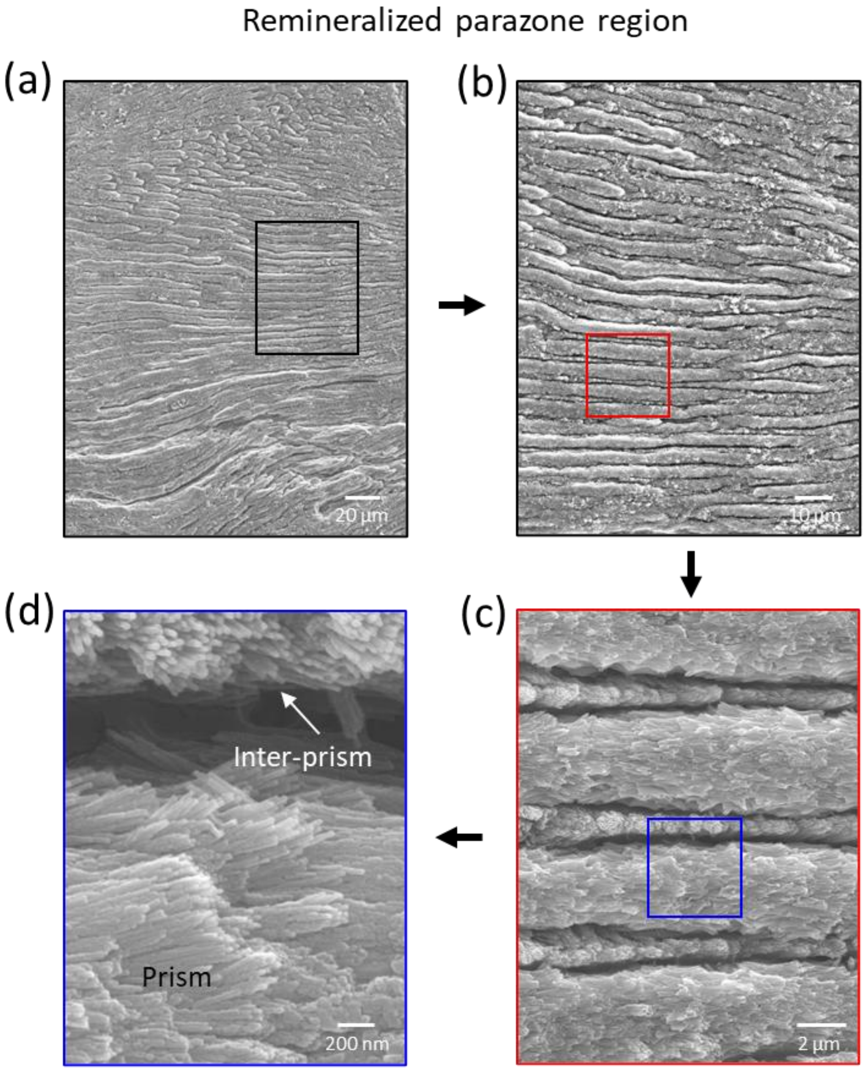


**Supplementary Fig. 22. Remineralized parazone regions of enamel.**
Low to high magnification SEM images showing remineralized **(a, b, c, d)** parazone regions of enamel. These results demonstrate the uniformity of the mineralized layer over large and uneven surfaces of enamel. For **(a - d)**, representative images from n = 8 independent experiments are used.

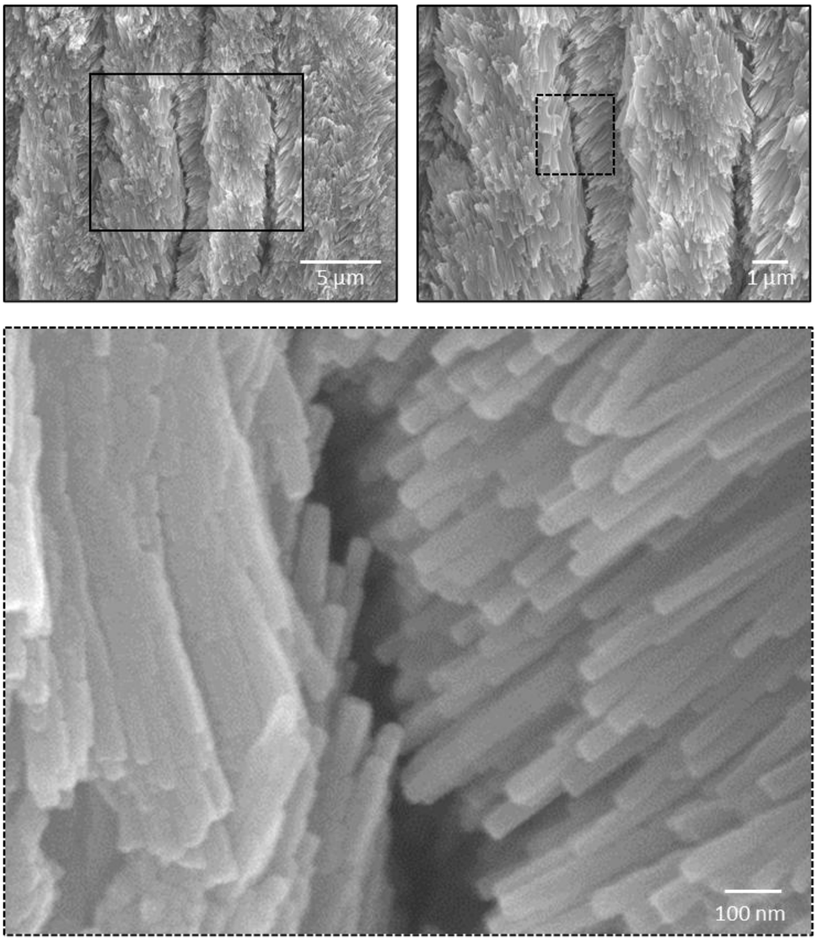


**Supplementary Fig. 23: High magnification SEM images confirm complete ELR degradation post elastase treatment.**

SEM images confirming the complete degradation of the ELR coating from parazone region prior to all mechanical and functional tests. ELR coating was degraded using elastase to expose the mineralized layer. Representative images from n = 8 independent experiments are used.

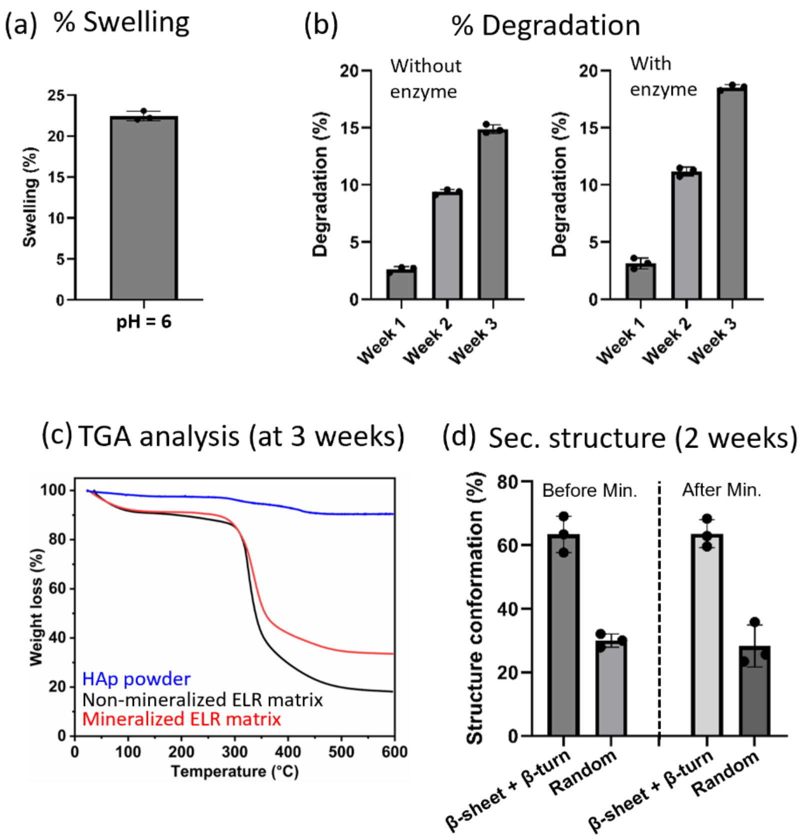


**Supplementary Fig. 24: Interplay between mineralization and degradation of the ELR matrices.** **(a)** Swelling property of the ELR membranes at pH = 6. **(b)** Degradation studies of ELR membranes during mineralization using artificial saliva without and with salivary enzyme proteinase K (15 ng/mL). **(c)** Thermogravimetric analysis (TGA) and **(d)** FTIR analyses of the ELR membranes before and after mineralization. Sample size of n = 3 was used for **(a, b, and d)**.

(a)

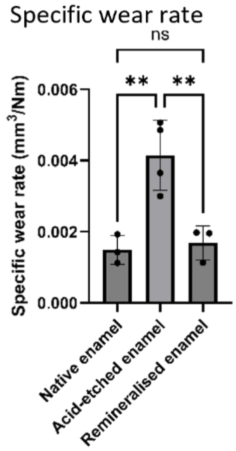


(b) W = WS X V (1)

Therefore, WS = W/V

W = CoF x F x L (2)

We know that specific wear rate (SWR) is expressed as V/(F x L),

Therefore, WS = CoF/SWR (3)

**Supplementary Fig. 25: Specific wear rate and wear strength calculation for remineralized enamel.** **(a)** Specific wear rate (SWR) of native, acid-etched, and remineralized prismatic enamel (n = 3 samples for each group). **(b)** Assuming the Reye hypothesis, the volume 'V' of removed material is proportional to the work done 'W' by the frictional force and the constant of proportionality is the wear strength (WS). F = normal load and L = sliding length. Data are presented as mean ± SD. Statistical significance was analysed using two-sided one-way ANOVA (Tukey test) in GraphPad Prism ver. 10. In **(a)** ** represents p = 0.0050 between native and acid-etched enamel, ** represents p = 0.0075 between acid-etched and remineralized enamel, and 'ns' represents no significant difference.

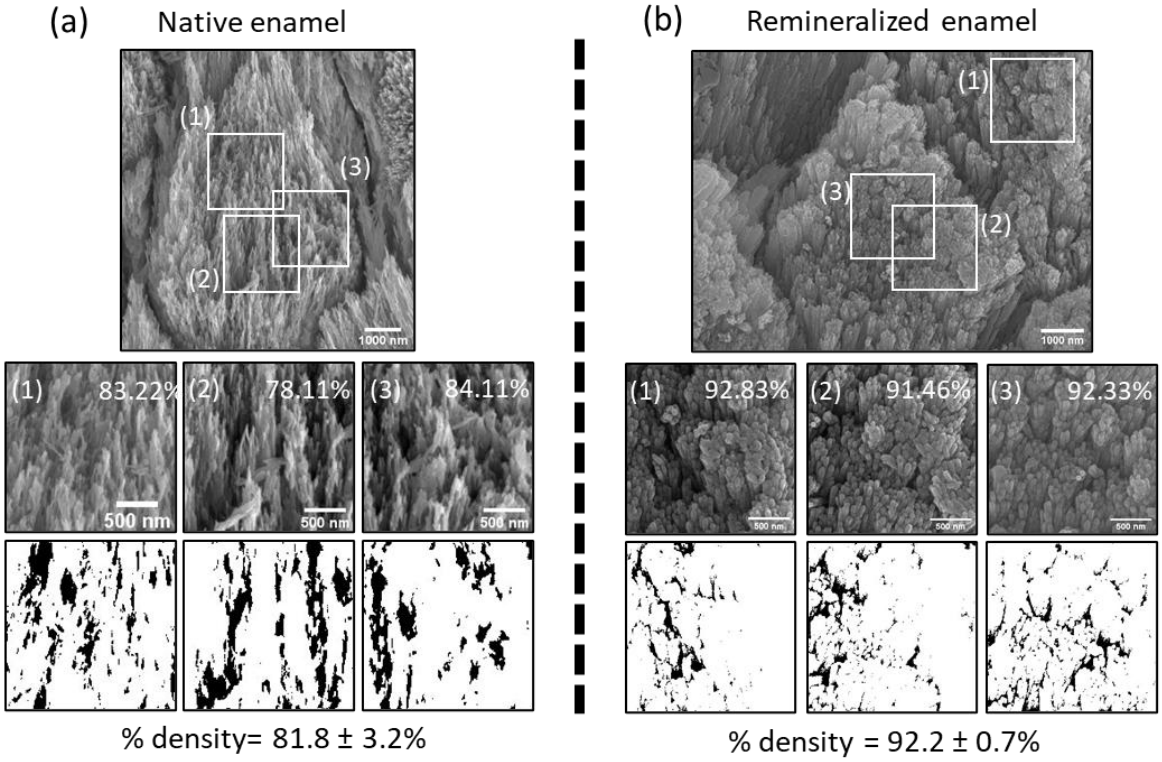


(c) Table: Calculation of the wear strength (WS) of remineralized enamel based on crystal density (%) and WS of native enamel (153.9 GPa) using following expression:

$$\frac{\%density_{(Remin)}}{\%density_{(Native)}} = \frac{WS_{(Remin)}}{WS_{(Native)}} \quad (1)$$

| % density (Native) | % density (Remineralized) | Wear strength (Remineralized) (GPa) |
|---|---|---|
| Area 1 = 83.22 | Area 1 = 92.83 | (92.83/83.22)*153.9 = 171.67 |
| Area 2 = 78.11 | Area 2 = 91.46 | (91.46/78.11)*153.9 = 180.20 |
| Area 3 = 84.11 | Area 3 = 92.33 | (92.33/84.11)*153.9 = 168.94 |
| | Average $WS_{(Remin)}$ | 173.6 |
| | Std. dev. | 5.8 |

**Supplementary Fig. 26: Estimation of the crystal density and its co-relation with Wear strength (WS)**.

Calculation of %density of **(a)** native and **(b)** remineralized enamel prism using ImageJ software. **(c)** Calculated WS of remineralized enamel using %density co-related well with the WS calculated using coefficient-of-friction (CoF) and specific wear rate (SWR) values.

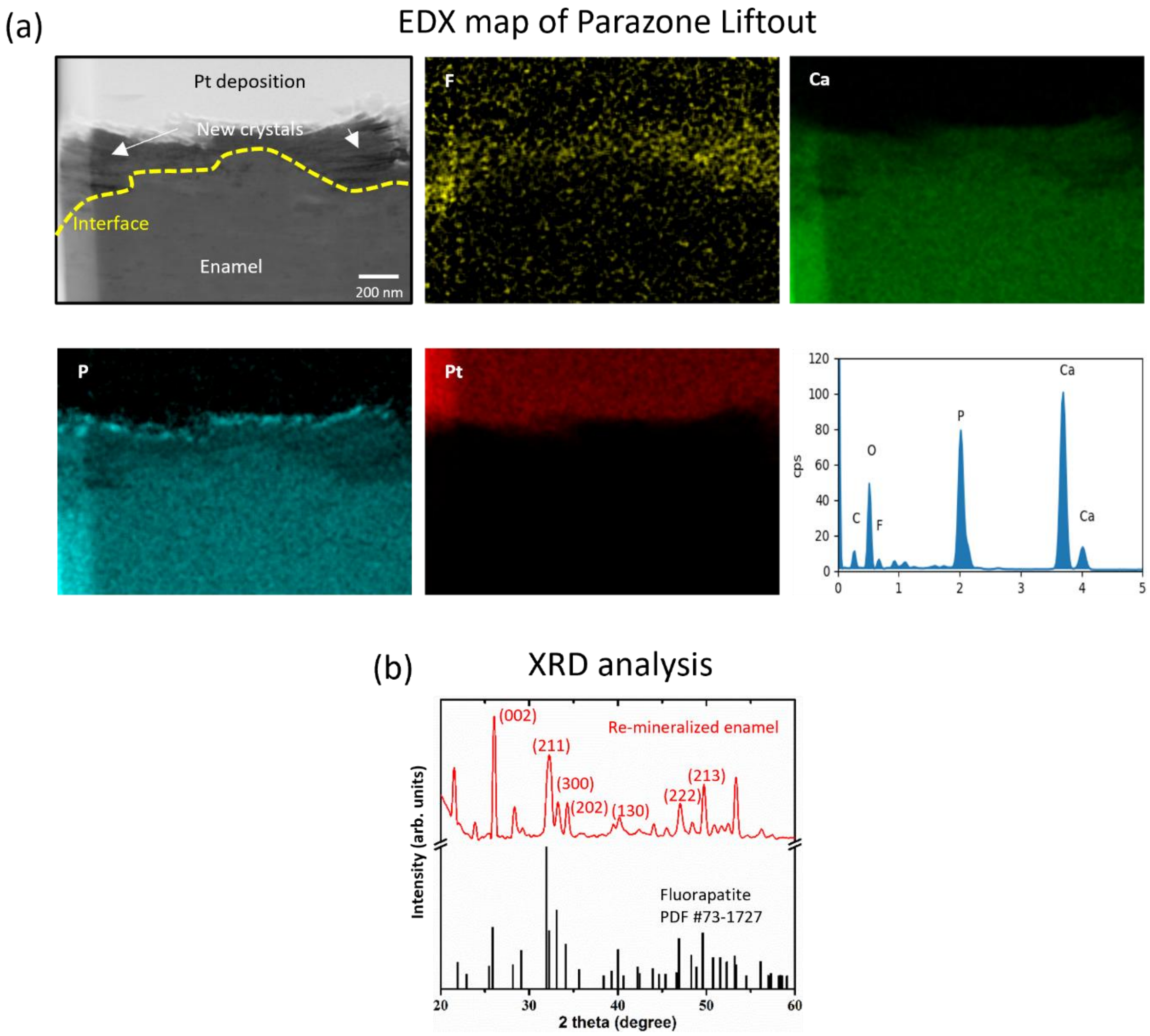


**Supplementary Fig. 27: EDX and XRD analysis of remineralized enamel.**
**(a)** EDX mapping of FIB-TEM sample prepared form parazone region confirmed the formation of flourapatite (FAp) nanocrystals when mineralized using solution supersaturated with respect to FAp. **(b)** XRD analysis of remineralized enamel further confirmed formation of FAp.

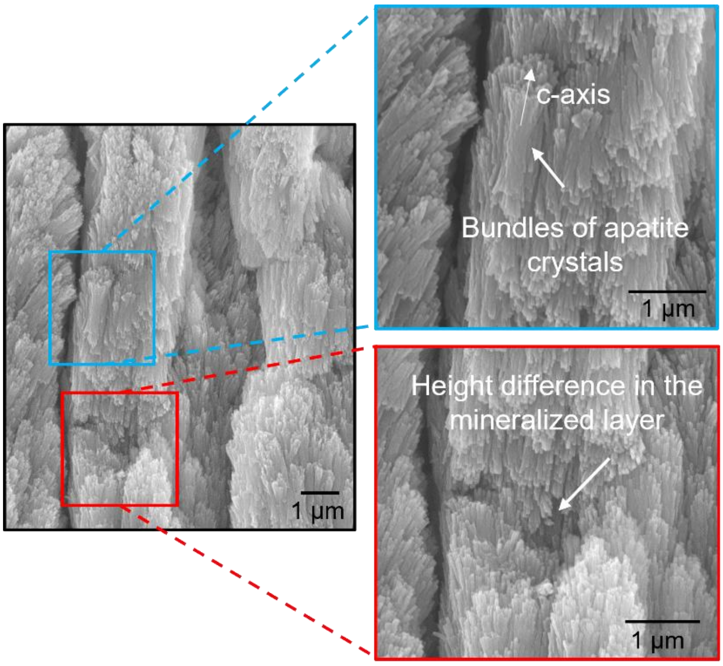


**Supplementary Fig. 28: SEM images of remineralized parazone region of enamel.**
SEM images of remineralized parazone prisms showing bundles of apatite nanocrystal nucleating on the a-axis while extending and growing along the c-axis. Representative images from n = 8 independent experiments are used.

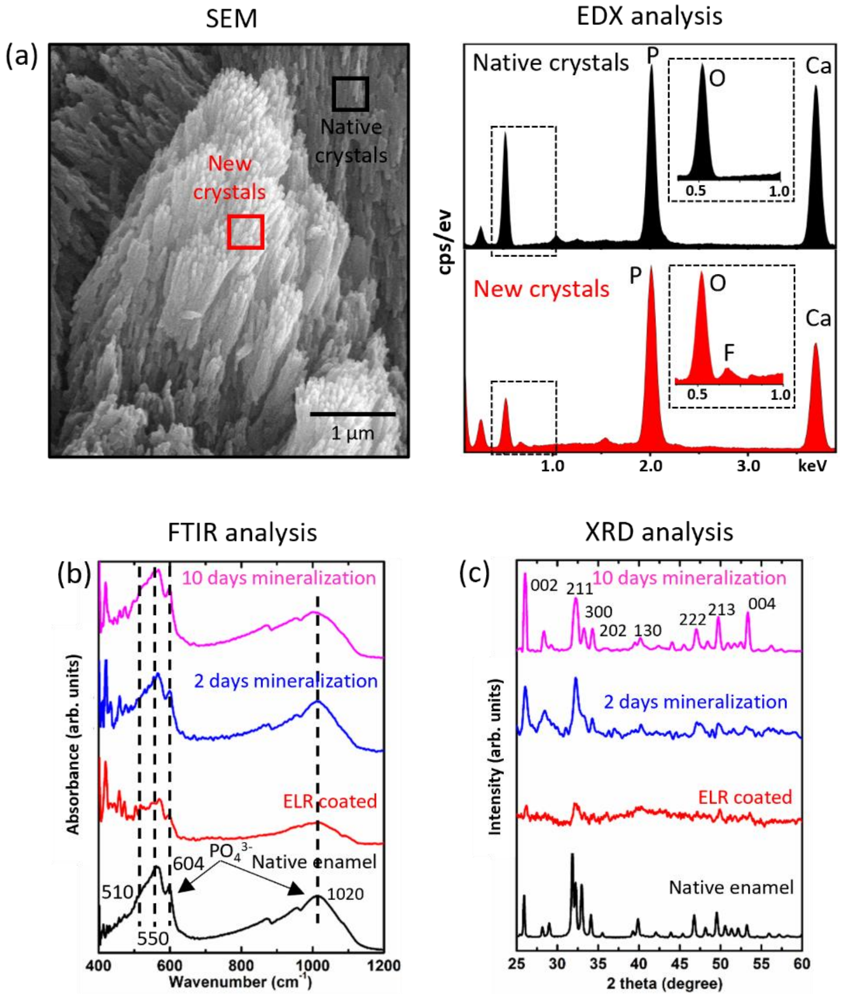


**Supplementary Fig. 29: EDX analysis confirms FAp formation on parazone region of enamel.**
**(a)** SEM image (representative image from n = 8 samples) and EDX spectra of partially remineralized diazone prism. EDX spectrum from remineralized enamel confirmed formation of FAp in contrast to CHAp of native enamel. **(b)** FTIR and **(c)** XRD spectra of native and remineralized enamel samples.

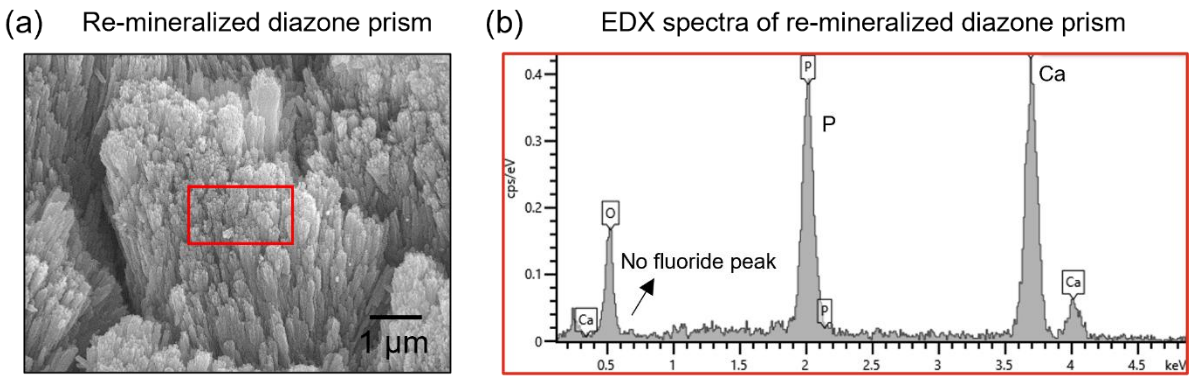


**Supplementary Fig. 30: HAp formation using mineralization solution devoid of $F^-$ ions.**
**(a)** SEM image (representative images from n = 3 samples) and **(b)** EDX spectra of diazone prism remineralized using mineralization solution devoid of $F^-$ ions.

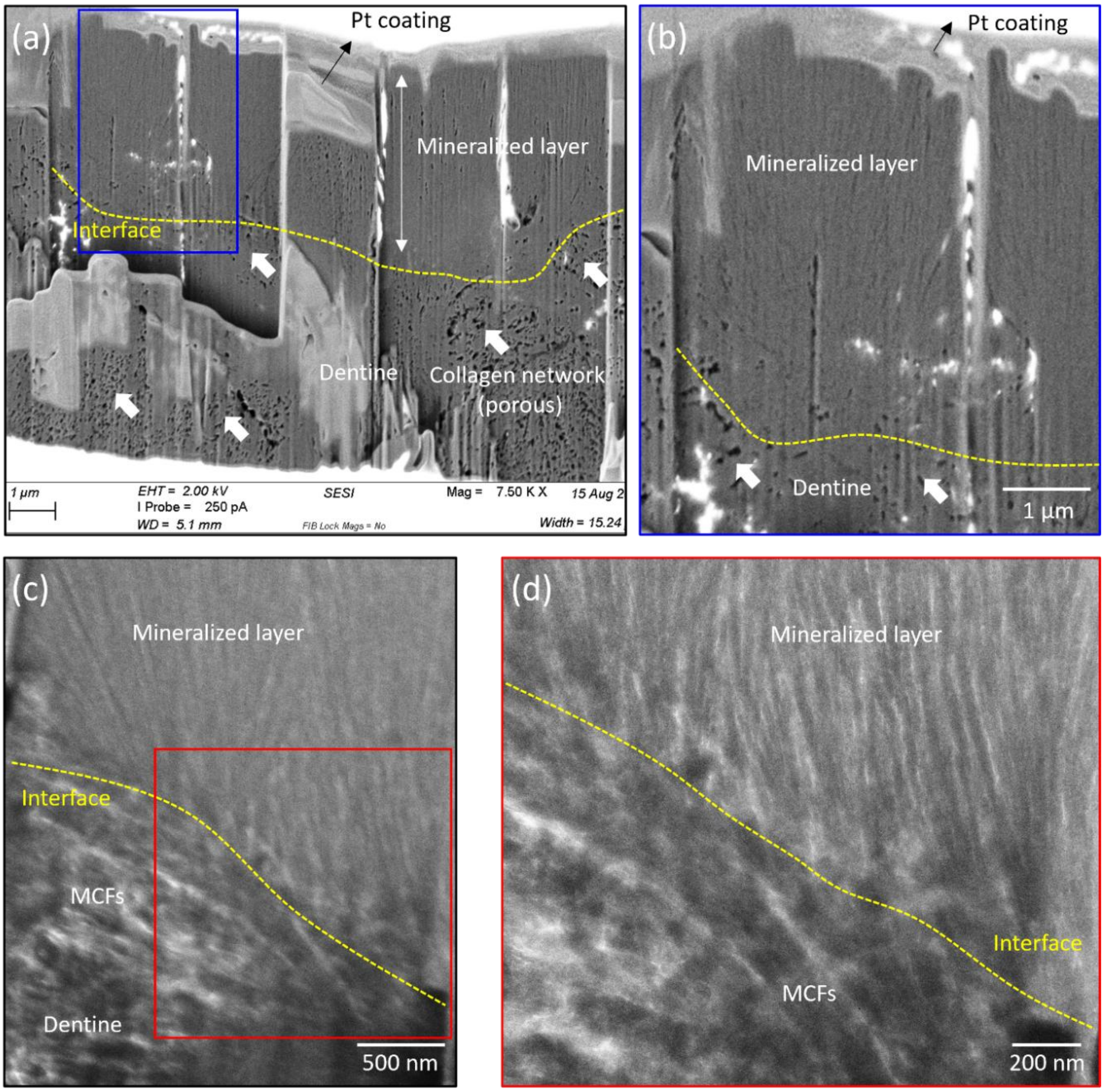


**Supplementary Fig. 31. Integration between the mineralized layer and mineralized collagen fibrils (MCFs) at interface.**

**(a, b)** Low magnification FIB-SEM images showing cross-section of the mineralized layer grown over dentine surface. White arrows point to the porous-looking dentine formed by 3D collagen network. **(c)** TEM image showing the cross-section of the integrated mineralized layer and dentine at interface. **(d)** TEM image showing integration between the mineralized layer and MCFs of dentine. For **(a, c)**, representative images from n = 3 samples are used.

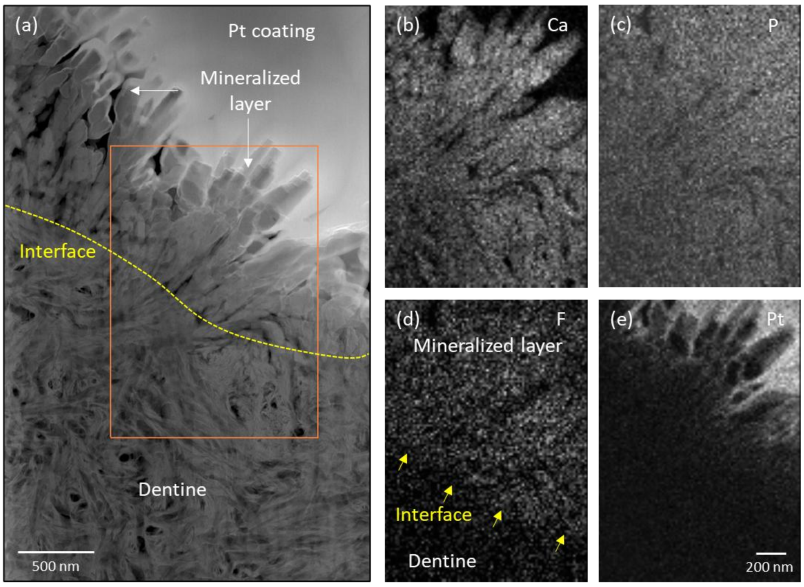


**Supplementary Fig. 32: TEM-EDX mapping of the mineralized dentine surface.**
**(a)** TEM micrograph showing mineralized layer grown on dentine surface. EDX maps showing **(b)** Ca, **(c)** P, **(d)** F, and **(e)** Pt signals from the mineralized layer and dentine interface. Presence of F signal from the fluorapatite crystals grown on dentine surface distinctly differentiates between native dentine and mineralized layer.

**Description:** EDX mapping revealed a distinct fluorine (F) signal arising from the newly grown fluorapatite crystals (**Supplementary Fig. 32**) while no F signal was detected in the native dentine region, thus differentiating the interface between the native and remineralized regions.

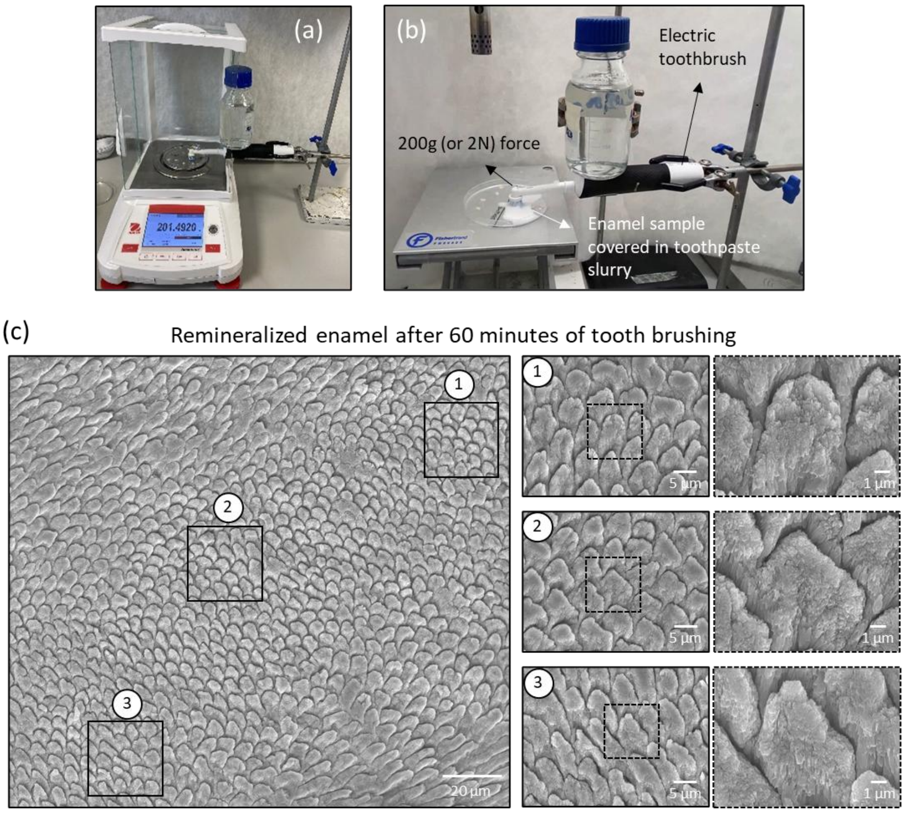


**Supplementary Fig. 33: Customized setup to simulate tooth brushing.**
**(a)** Setup to generate 200g or 2N force. **(b)** Sections of remineralized enamel were brushed for different time periods (15 and 60 minutes) using an electric tooth brush. 2N force was applied by the toothbrush tip onto the sample which was covered in the toothpaste slurry. **(c)** SEM images of remineralized prismatic enamel after 60 minutes of continuous brushing (n = 3 samples).

*Estimation of the number of days of tooth brushing simulated by continuously brushing for different time periods.*
American Dental Association (ADA)* recommends to brush teeth for two minutes twice a day i.e., 240 seconds of toothbrushing everyday

- Typically most adults have 32 teeth.
- Therefore, time spent on brushing individual tooth everyday is 240/32 = 7.5 seconds or ~8 seconds
- Thus, 15 minutes of continuous brushing on enamel section is equivalent to (15 x 60)/8 = 112.5 days of toothbrushing
- Likewise, 60 minutes of continuous brushing on enamel section is equivalent to (60 x 60)/8 = 450 days of toothbrushing

*https://www.ada.org/resources/research/science-and-research-institute/oral-health-topics/toothbrushes

**Description:** Low magnification SEM imaging at 3 different regions of the same sample demonstrates that the mineralized layer does not exhibit any visual microstructure loss after brushing for 60 minutes. This 60-minute time period of continuous brushing mimics tooth brushing for 1 year in a real scenario (**Supplementary Fig. 33**).

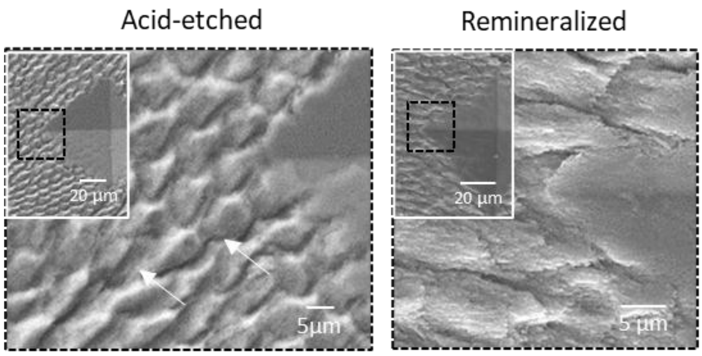


**Supplementary Fig. 34:** SEM images of acid-etched and remineralized prismatic enamel after indentation (n = 3 samples). White arrows in acid etched enamel sample indicate crack formation.

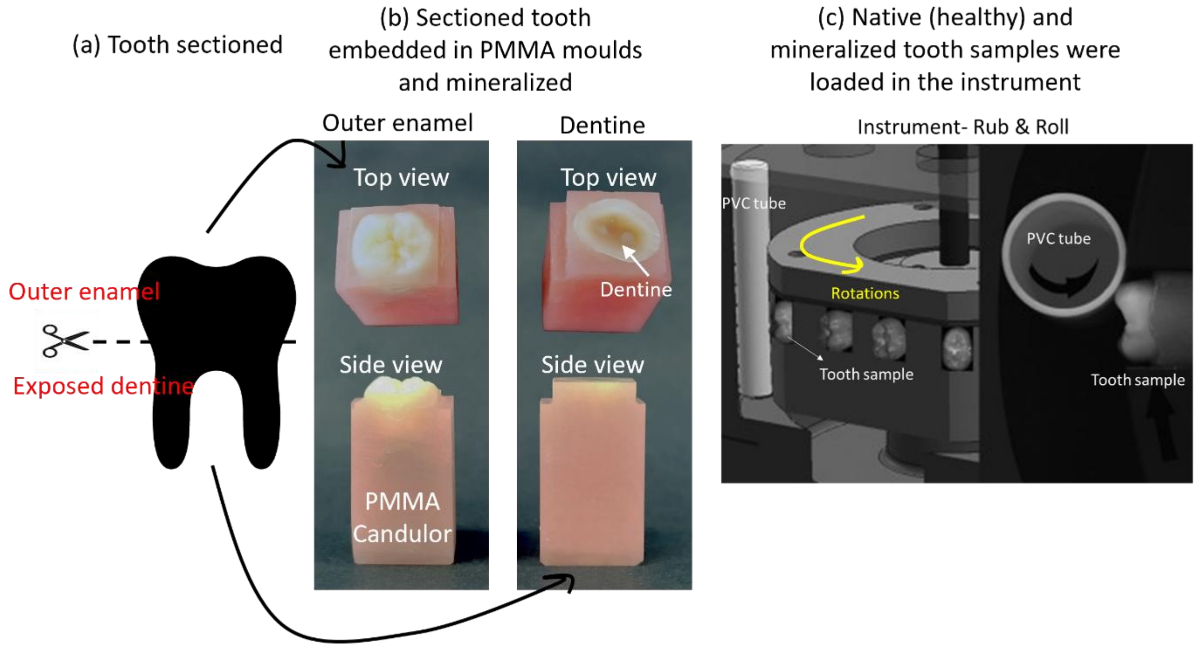


**Supplementary Fig. 35: Setup to simulate tooth abrasion.**
**(a)** Molar tooth samples were sectioned 1 mm above the cemento–enamel junction. **(b)** Samples were embedded in dental acrylic resin (PMMA Candulor), exposing the enamel and/or dentine surface. **(c)** Enamel and dentine samples were mounted on a rotating cylinder, where the samples protruding 1 mm above the cylinder surface and 4 rods with 4 PVC tubes are mounted where the loading is about 75N. The specimens are exposed to cyclic loading with water. A load of 75 N was applied for a continuous rotations of 2 weeks with a rotation speed of 10 RPM. A total of 806400 cycles were performed that imitated about 3.5 years of clinical loading. (c) is adapted from Ref.[25]. Copyright (2014), with permission from Elsevier.

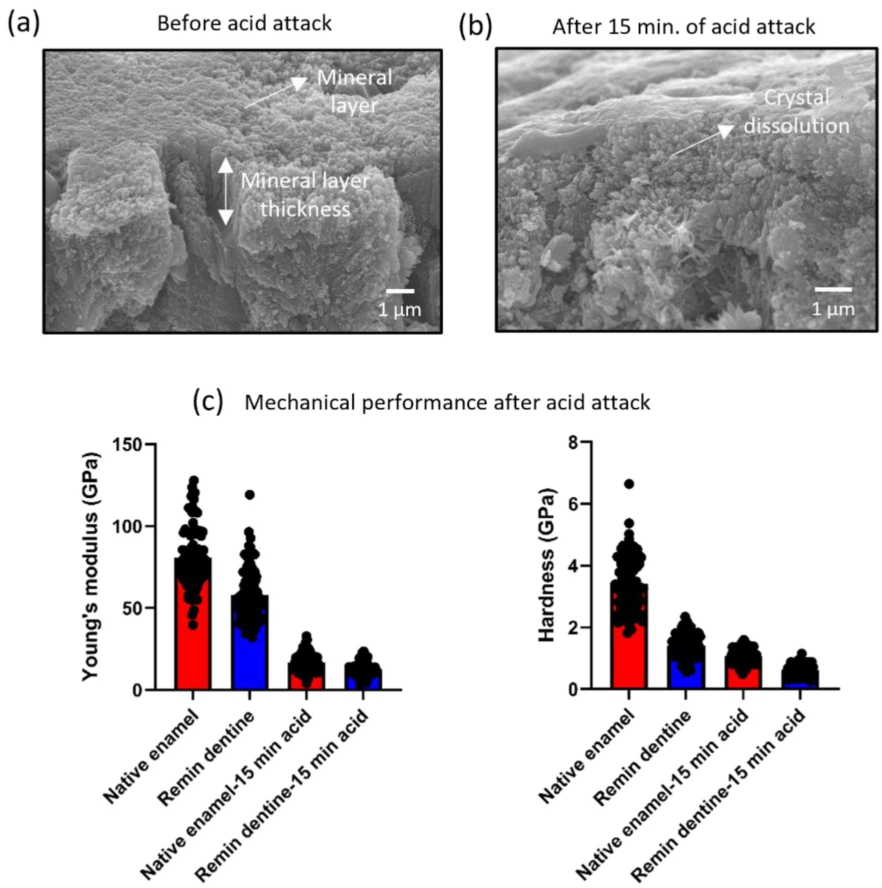


**Supplementary Fig. 36: Surface mineralization protects dentine against acid attack.**
SEM images of mineralized dentine surface **(a)** before and **(b)** after acid attack of 15 minutes (n = 3 samples). **(c)** Comparison of the mechanical properties between mineralized dentine and native enamel before and after acid attack of 15 minutes. In **(c)** bars represent the mean values of Young's modulus and hardness while dot plot represents indentation measurements compiled from 3 independent experiments for each group

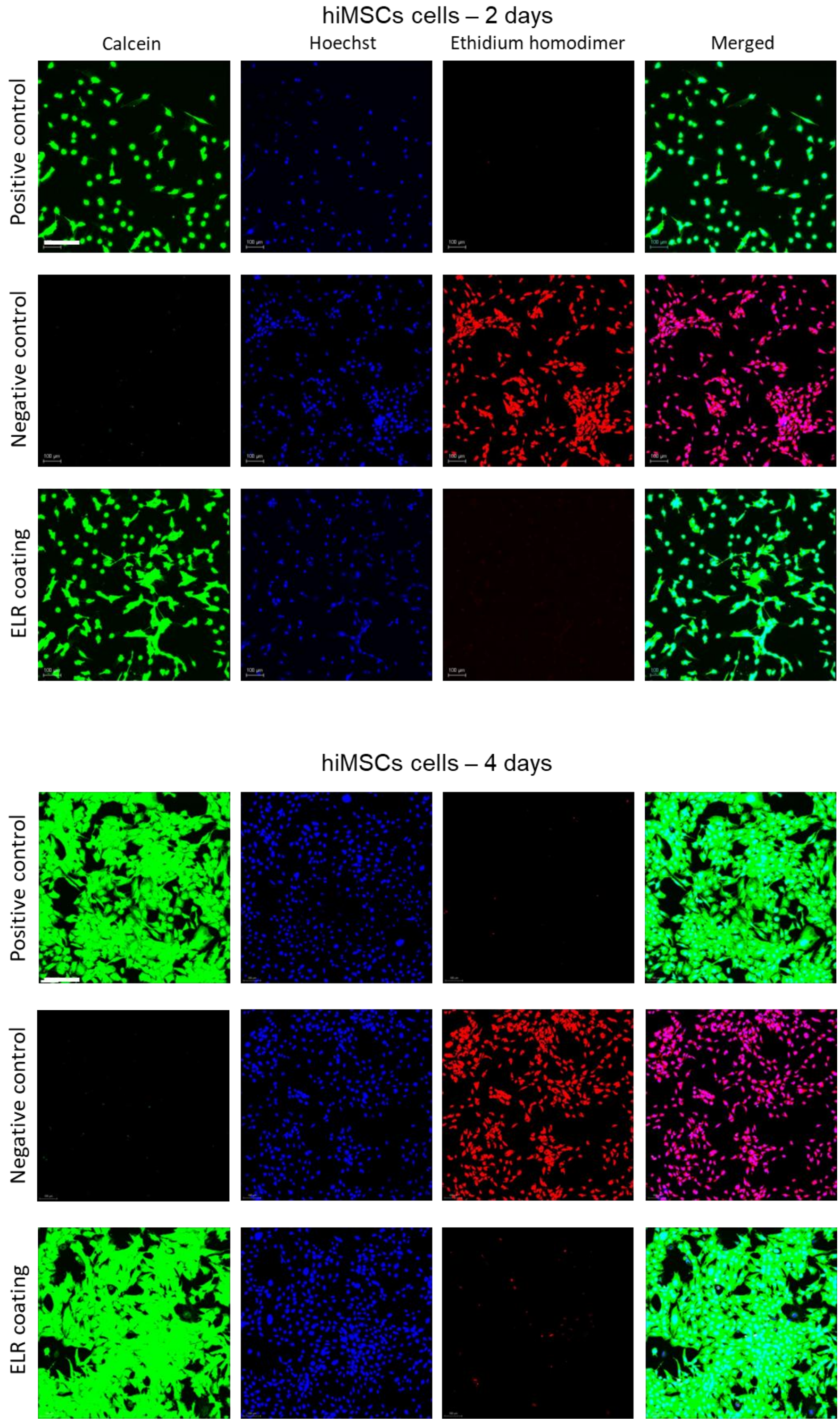


**Supplementary Fig. 37: Live/dead staining of human immortalised mesenchymal stem cells (hiMSCs).**

Confocal images showing hiMSCs cells cultured for 2 and 4 days on tissue culture treated plastic (n = 3) or ELR coated glass coverslips (n = 3). For positive control, cells were cultured on tissue culture treated plastic; for negative control, cells were cultured on tissue culture treated plastic but were lysed by treating with 100% DMSO for 5 minutes; and for ELR sample, cells were cultured on ELR coated glass coverslips. Scale bar = 200 µm.

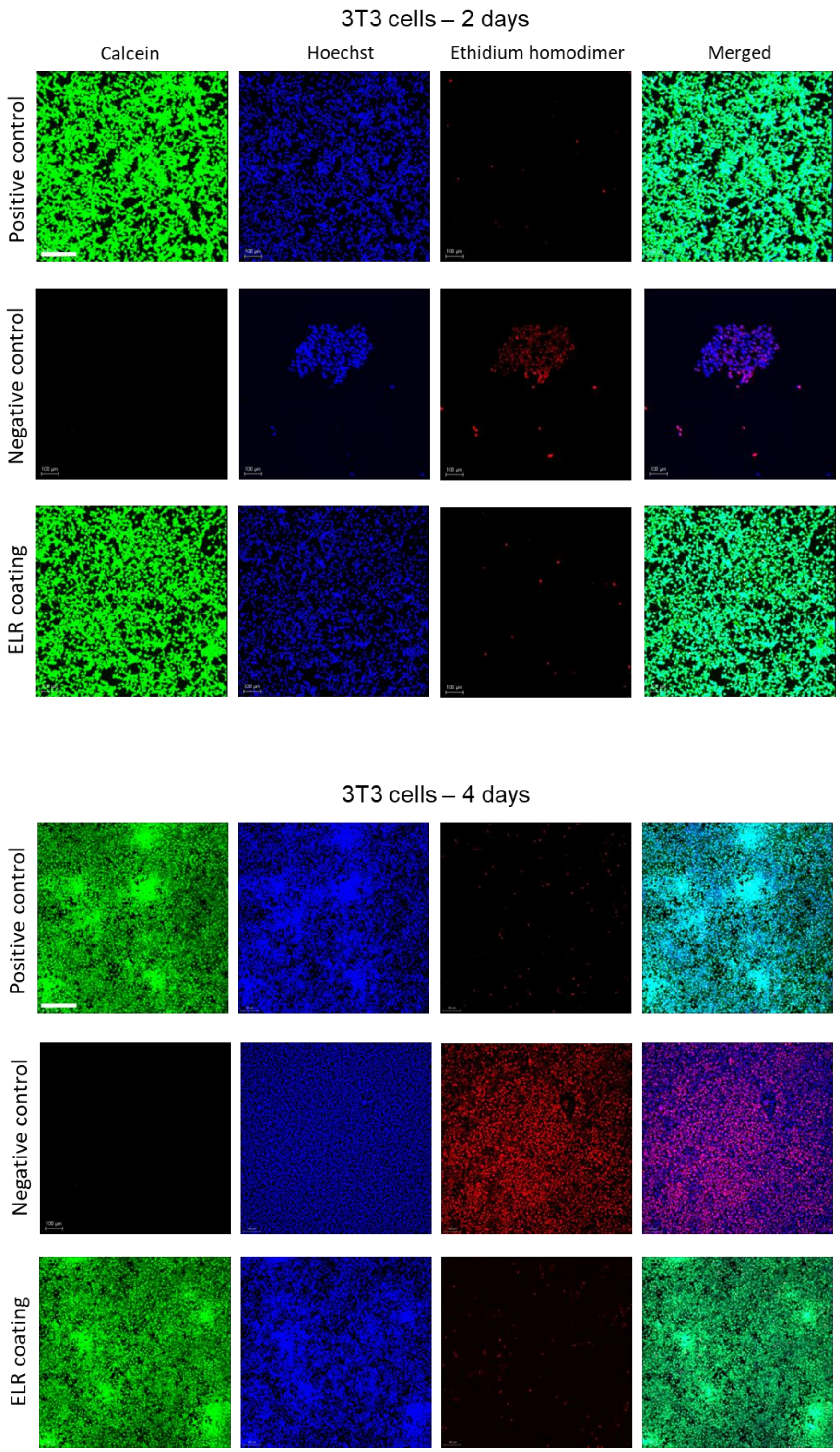


**Supplementary Fig. 38: Live/dead staining of mouse fibroblast 3T3 cells.**
Confocal images showing 3T3 cells cultured for 2 and 4 days on tissue culture treated plastic (n = 3) and ELR coated glass coverslips (n = 3). For positive control, cells were cultured on tissue culture treated plastic; for negative control, cells were cultured on tissue culture treated plastic but were lysed by treating with 100% DMSO for 5 minutes; and for ELR sample, cells were cultured on ELR coated glass coverslips. Scale bar = 200 µm.

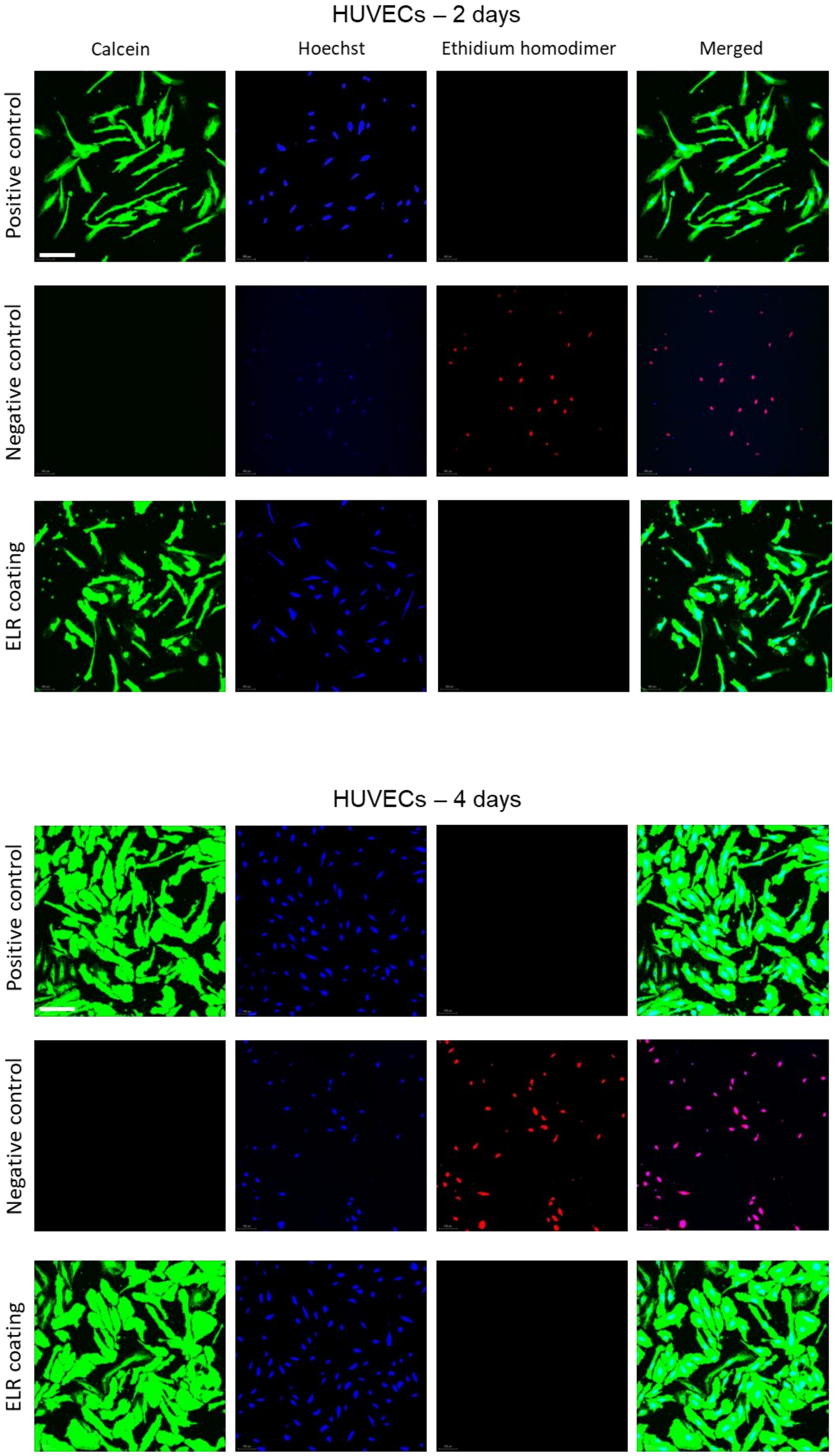


**Supplementary Fig. 39: Live/dead staining of human umbilical vein endothelial cells (HUVECs).** Confocal images showing HUVECs cultured for 2 and 4 days on tissue culture treated plastic (n = 3) and ELR coated glass coverslips (n = 3). For positive control, cells were cultured on tissue culture treated plastic; for negative control, cells were cultured on tissue culture treated plastic but were lysed by treating with 100% DMSO for 5 minutes; and for ELR sample, cells were cultured on ELR coated glass coverslips. Scale bar = 200 µm.

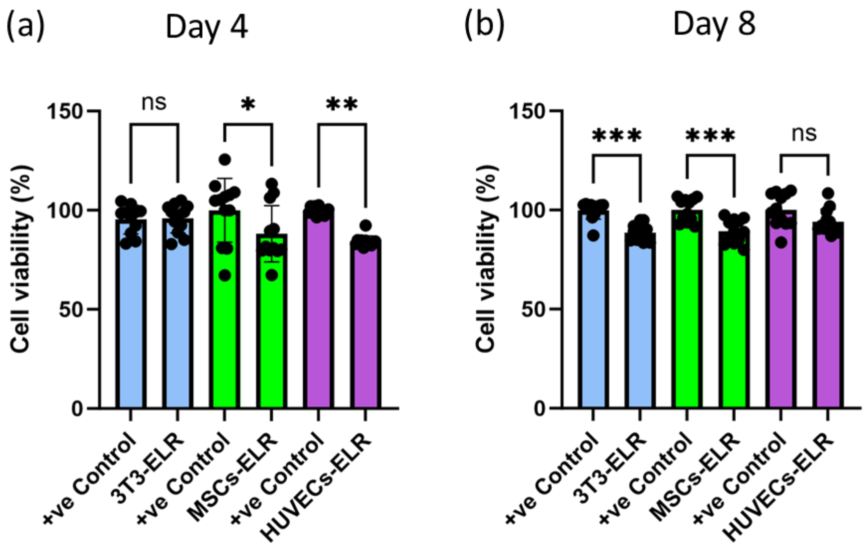


**Supplementary Fig. 40: Cell viability determination by MTS assay.**
Graphs showing comparision in the % cell viability after **(a)** 4 days and **(b)** 8 days of culturing different cells (3T3, hiMSCs, and HUVECs) on tissue culture treated plastic (+ve control, n = 6) and ELR coated glass coverslips (n = 6 samples). Data are presented as mean ± SD. Statistical significance was analysed using two-sided one-way ANOVA (Tukey test) in GraphPad Prism ver. 10. In **(a)** **, *, and ns represent significant difference p = 0.0029, 0.0478, and not significant. In **(b)** *** between +ve control and 3T3-ELR represents p = 0.0002, *** between +ve cntrol and MSC-ELR represents p = 0.0007, and 'ns' represents no significant difference.

**Discussion:** A colorimetric cell viability MTS assay was carried out after day 4 and 8 of cell seeding. These results demonstrated 85% or more cell viability for all 3 cell lines and is significantly higher compared to 70% viability recommended by ISO standard (10993-5) for materials to be considered cytocompatible[26].

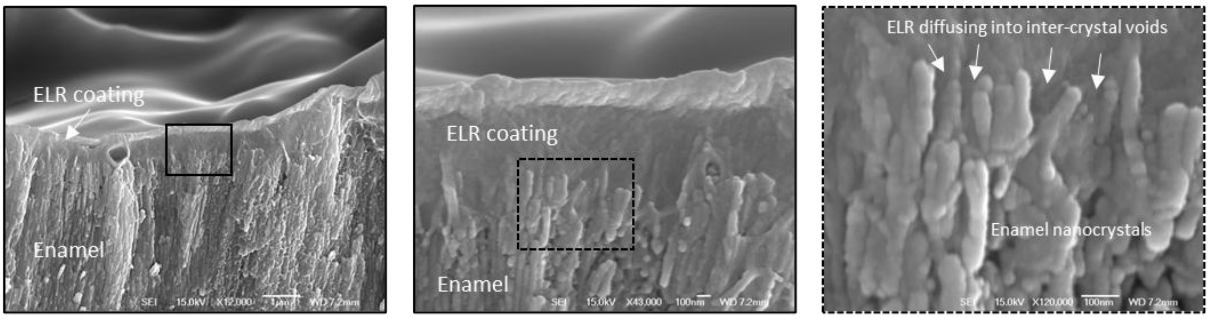


**Supplementary Fig. 41: ELR matrix diffuses deep into the inter-crystal voids on enamel surface.** SEM images showing the penetration of ELR solution into the inter-crystal voids and forming the ELR coating on enamel surface (representative images from n = 5 samples). ELR solution was prepared in ethanol/water solvent mixture (9/1 ratio) and comprised of 5% w/v ELR, 1.5 mM $Ca^{2+}$ ions, and 1.5% glutaraldehyde.

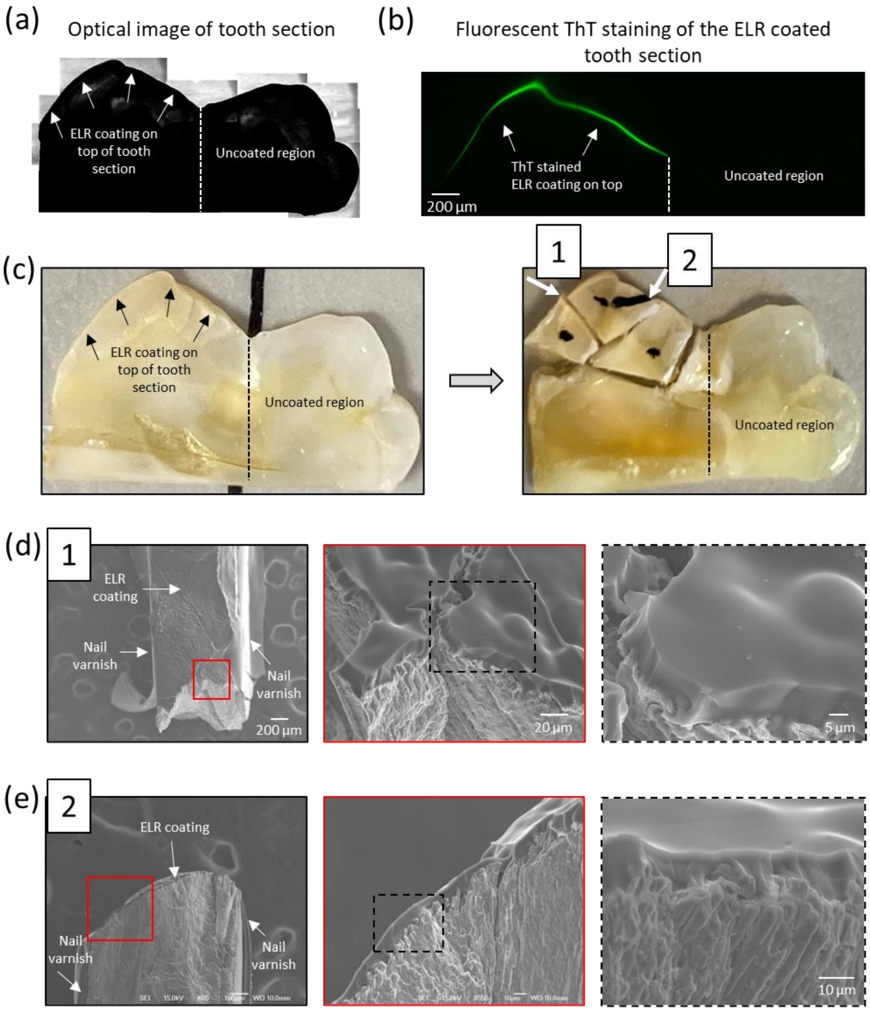


**Supplementary Fig. 42. Uniform ELR coating over large and convoluted tooth area.** **(a)** Optical image showing top of tooth section partially coated with ELR (left region) and the remaining with nail varnish. ELR coating was prepared using ethanol-water system and dried for 3-4 minutes under room temperature. **(b)** Fluorescent thioflavin T (ThT) staining of the ELR coated tooth section. ThT dye binds specifically to the β-sheet rich ELR coating (green colour) while uncoated region exhibits no staining. **(c)** Photographs showing ELR coated region of the tooth section that was chipped into 3 pieces. **(d, e)** SEM images confirm the uniform 10 µm thick ELR coating across the cross section of the chipped pieces (representative images from n = 3 independent experiments).

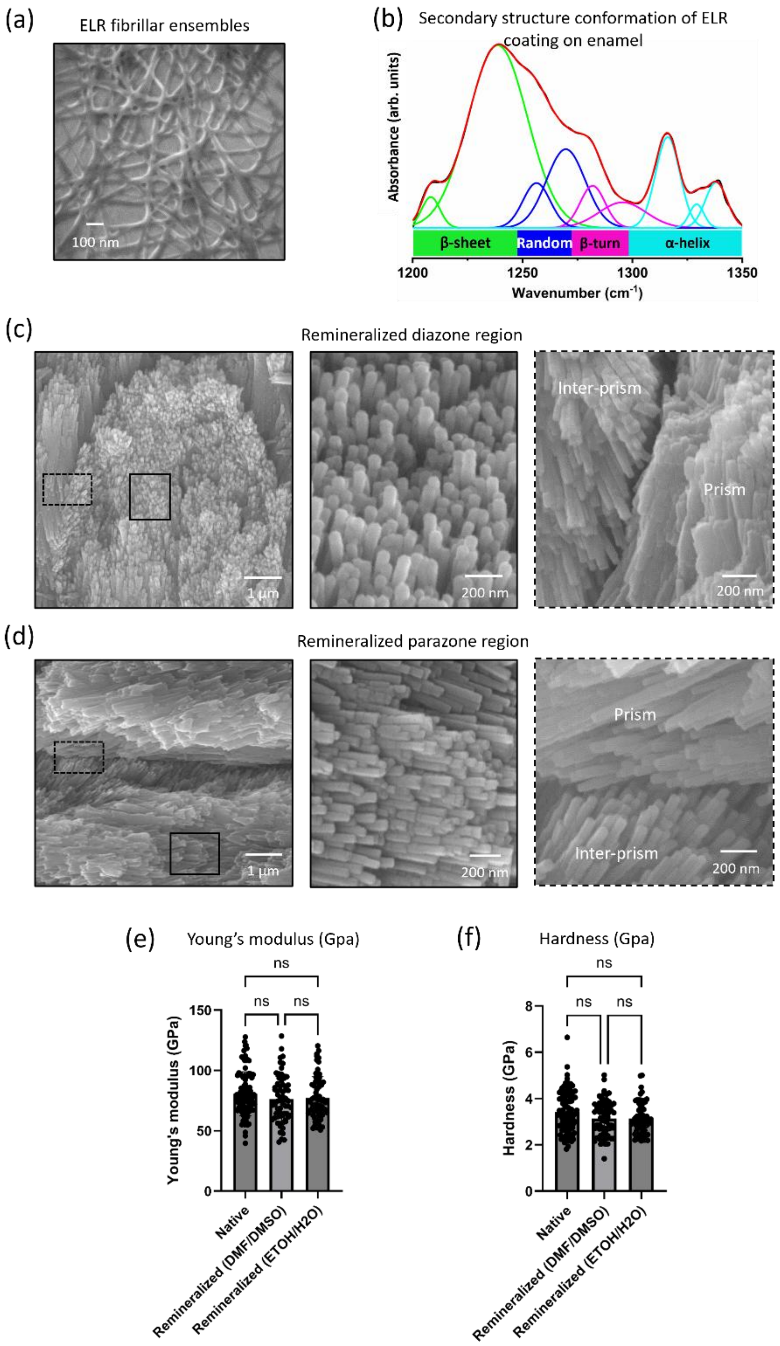


**Supplementary Fig. 43: Ethanol/water system forms ELR fibrillar ensembles, recreates microstructure, and restores mechanical properties.**

**(a)** SEM image showing ELR fibrillar ensembles synthesized using 1% w/v ELR in ethanol(ETOH)/water($H_2O$) mixture at a 9/1 ratio in presence of 1.5 mM $Ca^{2+}$ ions (n = 3 samples). **(b)** Secondary structure conformation of ELR coating on enamel. SEM images of **(c)** diazone and **(d)** parazone prisms remineralized within ELR matrix prepared using 5% w/v ELR dissolved in 9/1 ratio of ETOH/$H_2O$ mixture containig 1.5 mM $Ca^{2+}$ ions and 1.5% glutaraldehyde (representative mages from n = 8 independent experiments). Enamel samples remineralized using ETOH/$H_2O$ system exhibited similar **(e)** Young's modulus and **(f)** hardness compared to native enamel and enamel remineralized via DMF/DMSO system. In **(e and f)** bars represent the mean values of E and H while dot plot represents indentation measurements compiled from 3 independent experiments for each group. Statistical significance was analysed using two-sided one-way ANOVA (Tukey test) in GraphPad Prism ver. 10. ns represents not significant.

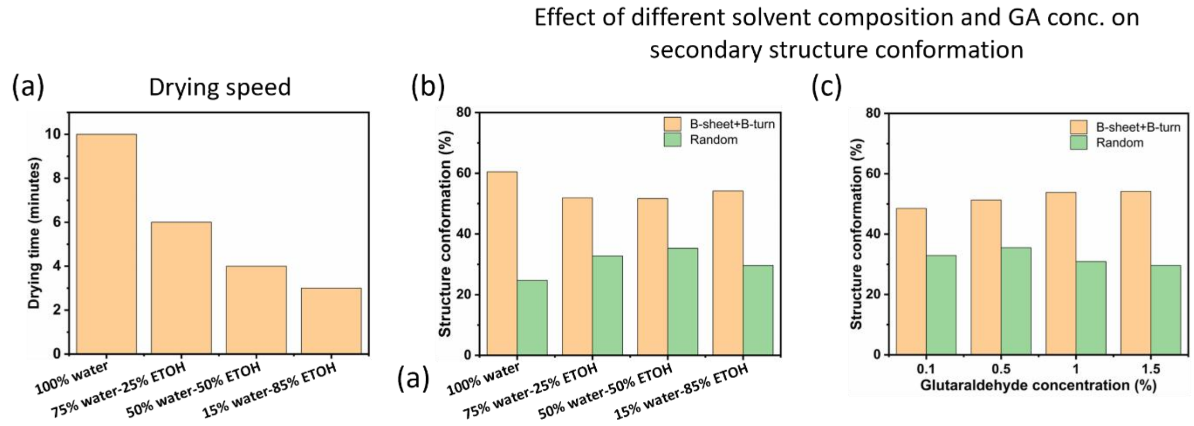


**Supplementary Fig. 44: Effect of solvent composition and crosslinker concentration on the secondary structure conformation of the ELR matrices.**

**(a)** Graph illustrating the reduction in drying time for ELR matrices as ethanol concentration increases. FTIR analysis confirms similar secondary structure conformation of ELR coatings independently of the **(b)** ethanol concentrations and **(c)** crosslinker concentrations.

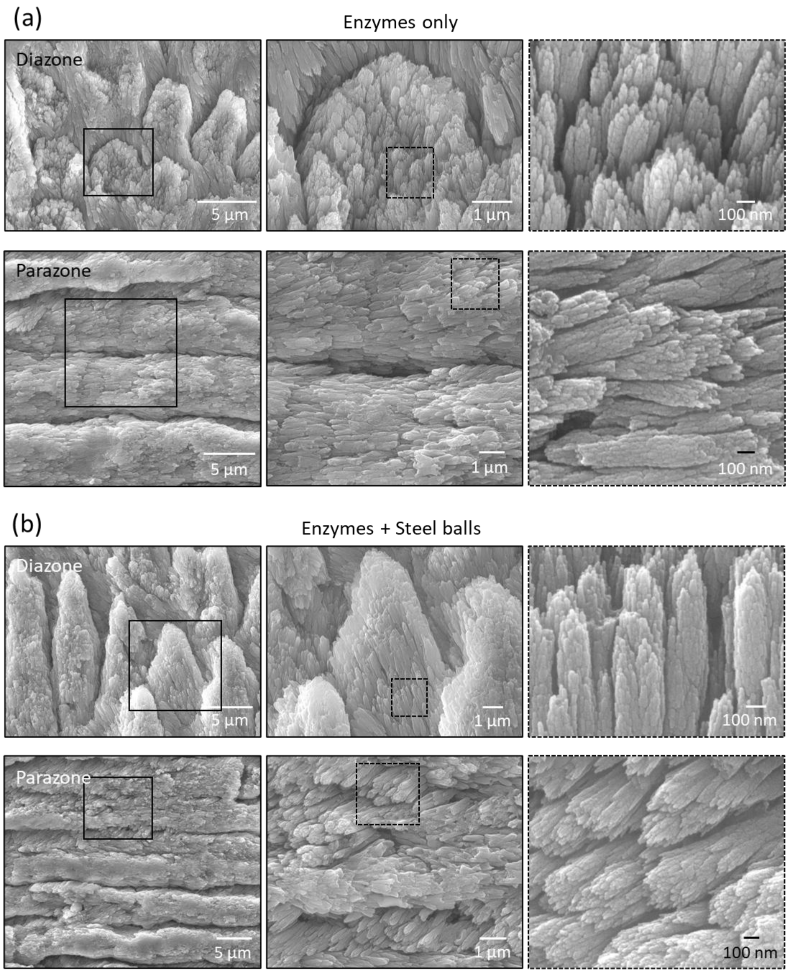


**Supplementary Fig. 45. Enamel remineralization under real oral environments.** SEM images showing prismatic enamel remineralized using modified-artificial saliva supplemented with **(a)** salivary enzymes and **(b)** salivary enzymes + steel balls to imitate real oral environments (i.e., both dynamic and abrasive conditions). Samples were vortexed at 1000 RPM at 37 °C for 10 days during mineralization. For **(a, b)**, representative images from n = samples are used.

**Discussion:** To demonstrate the functionality of our ELR coating under a real oral environment, we customized a specialized set-up comprising modified-artificial saliva (m-AS) by supplementing AS with different salivary enzymes such as α-amylase (100 U/mL), lysozyme (0.75 U/mL), and proteinase K (15 ng/mL). To imitate the dynamic and abrasive oral environment, we incubated the ELR coated enamel sections in the m-AS along with 5 steel balls with 2 mm diameter inside the 20 mL glass scintillation vials and stirred at 1000 RPM and 37 °C for 10 days. This methodology was adapted after slight modifications[27] to imitate external insults coming from food particles or tooth attrition, biting forces, etc. The results demonstrate that uniform remineralization of the enamel surface is achieved under these abrasive conditions.

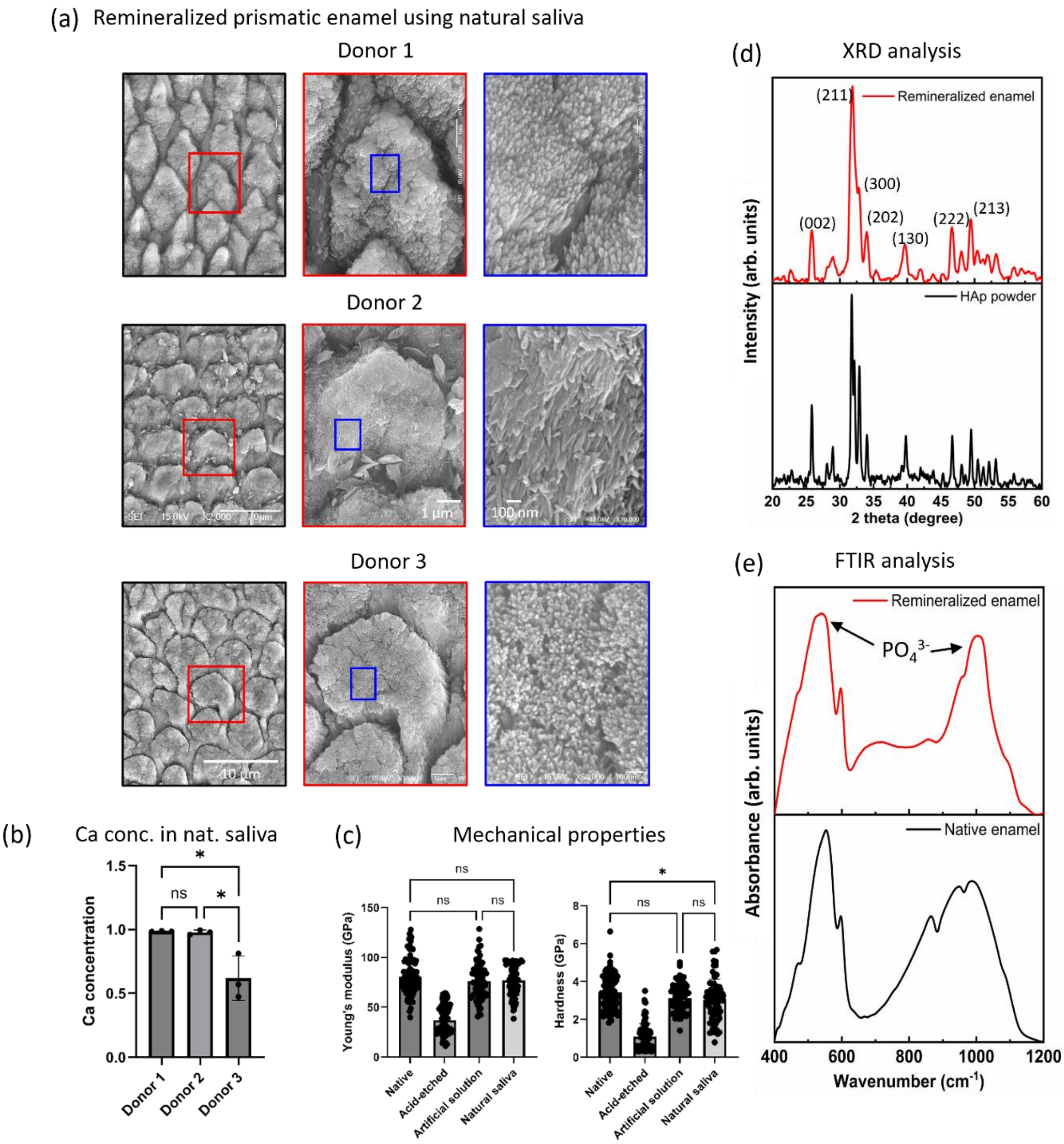


**Supplementary Fig. 46. Enamel remineralization using natural human saliva.**
**(a)** SEM images showing prismatic enamel remineralized using human saliva from 3 different donors (n = 3 samples). **(b)** Analysis of Ca ion concentration in the natural saliva collected from n = 3 different donors. **(c)** Mechanical properties, **(d)** XRD, and **(e)** FTIR analyses of enamel remineralized using natural saliva. In (c), bars represent the mean values of E and H while dot plot represents indentation measurements compiled from 3 independent experiments for each group. Statistical significance was analysed using two-sided one-way ANOVA (Tukey test) in GraphPad Prism ver. 10. In **(b)** * between Donor 2 and Donor 3 represents p = 0.0114 and in between Donor 1 and Donor 3 represents p = 0.0104. In **(c)** * represents p = 0.0124 and 'ns' represents not significant.

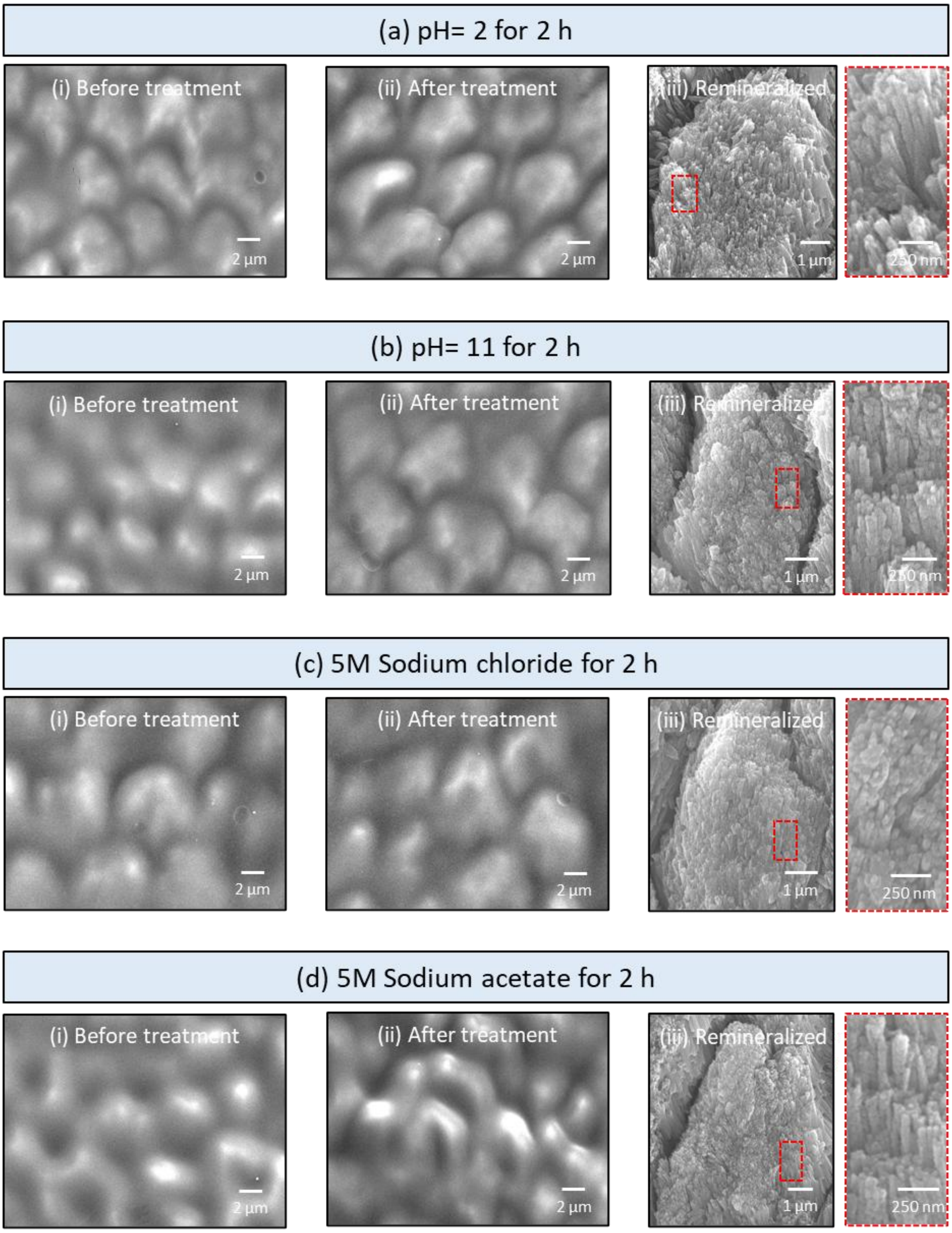


**Supplementary Fig. 47: ELR coating is highly stable against different chemical treatments.** SEM images showing ELR coatings on enamel sections before and after different treatments and remineralized enamel under different treatments **(a)** pH 2 for 2 h, **(b)** pH 11 for 2 h, **(c)** 5M sodium chloride for 2 h, and **(d)** 5M sodium acetate for 2 h. The SEM images of ELR coated enamel showing before and after treatment appear blurry due to a visual effect created by the ELR coating uniformly conforming over the enamel surfaces. For **(a to d)**, representative images from n = 3 samples are used.

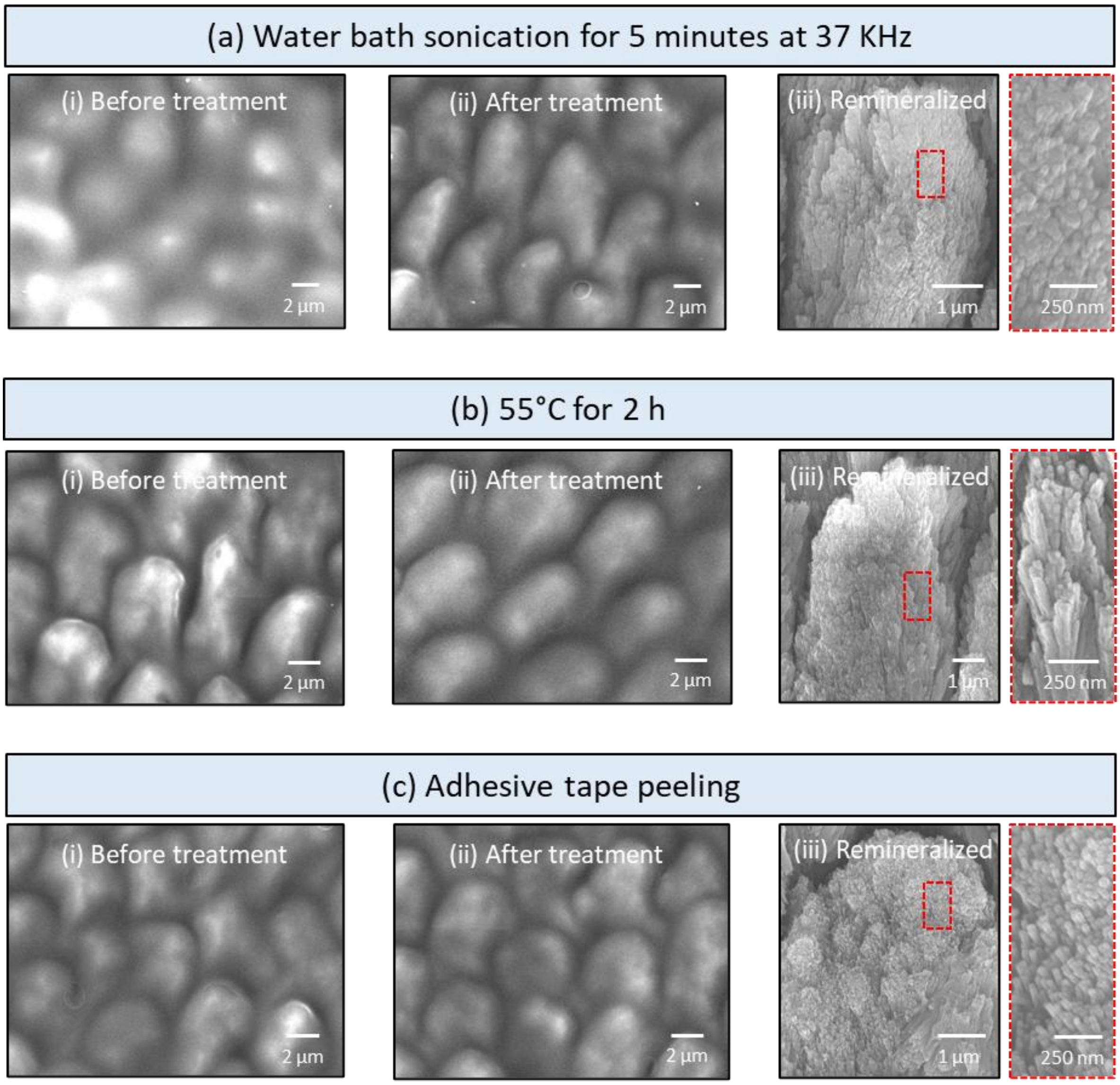


**Supplementary Fig. 48: ELR coating is highly stable against different chemical treatments.** SEM images showing before, after, and remineralized enamel under different treatments **(a)** water bath sonication for 5 min at 37 KHz **(b)** 55°C for 2 h, and **(c)** adhesive tape peeling. The SEM images of ELR coated enamel showing before and after treatment appear blurry due to a visual effect created by the ELR coating uniformly conforming over the enamel surfaces. For **(a to c)**, representative images from n = 3 samples are used.

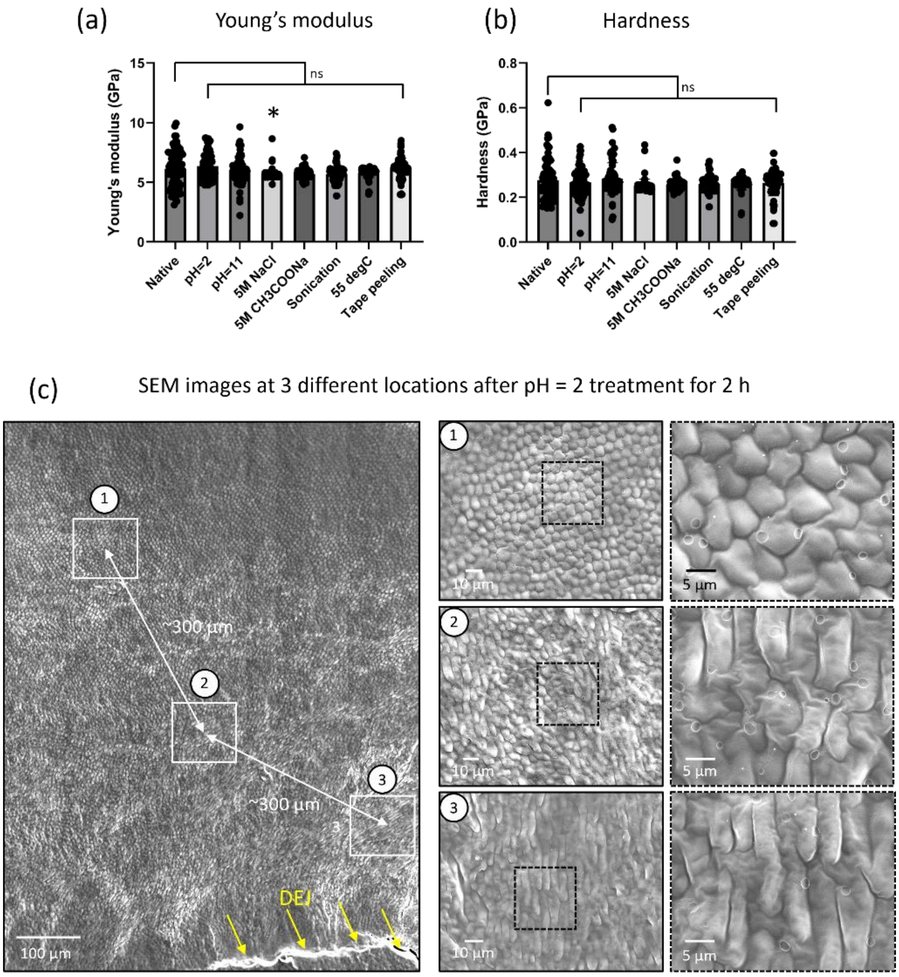


**Supplementary Fig. 49: Characterization of ELR coating after physical and chemical treatments.** Graphs showing mechanical properties i.e., **(a)** Young's modulus and **(b)** hardness of the ELR coatings before (native) and after physical and chemical treatments. **(c)** SEM images showing the morphology of the ELR coating at 3 different locations (each at least ~300 µm apart) after treatment at pH = 2 for 2 h. Bars represent the mean values of E and H while dot plot represents indentation measurements compiled from 3 independent experiments for each group. Data are presented as mean ± SD. Statistical significance was analysed using two-sided one-way ANOVA (Tukey test) in GraphPad Prism ver. 10. In **(a)** * between native and 5M NaCl represents p = 0.0226, and 'ns' represents no significant difference.

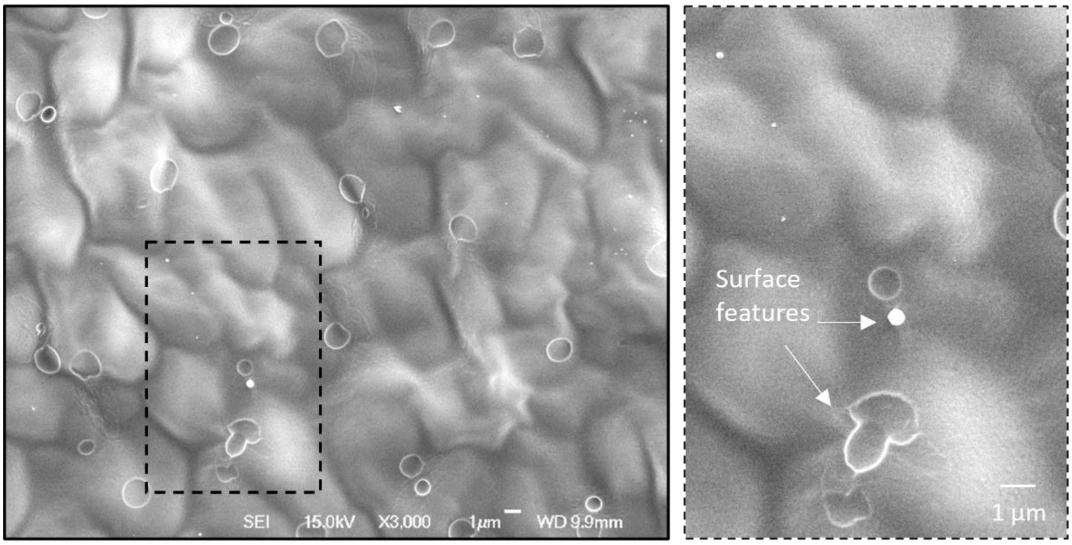


**Supplementary Fig. 50: SEM images of an ELR coated enamel.**
SEM images reveal sharp surface features on the ELR coating, though they appear blurry due to the visual effect of the coating uniformly conforming to the enamel surface. Representative images from n = 4 samples are used.

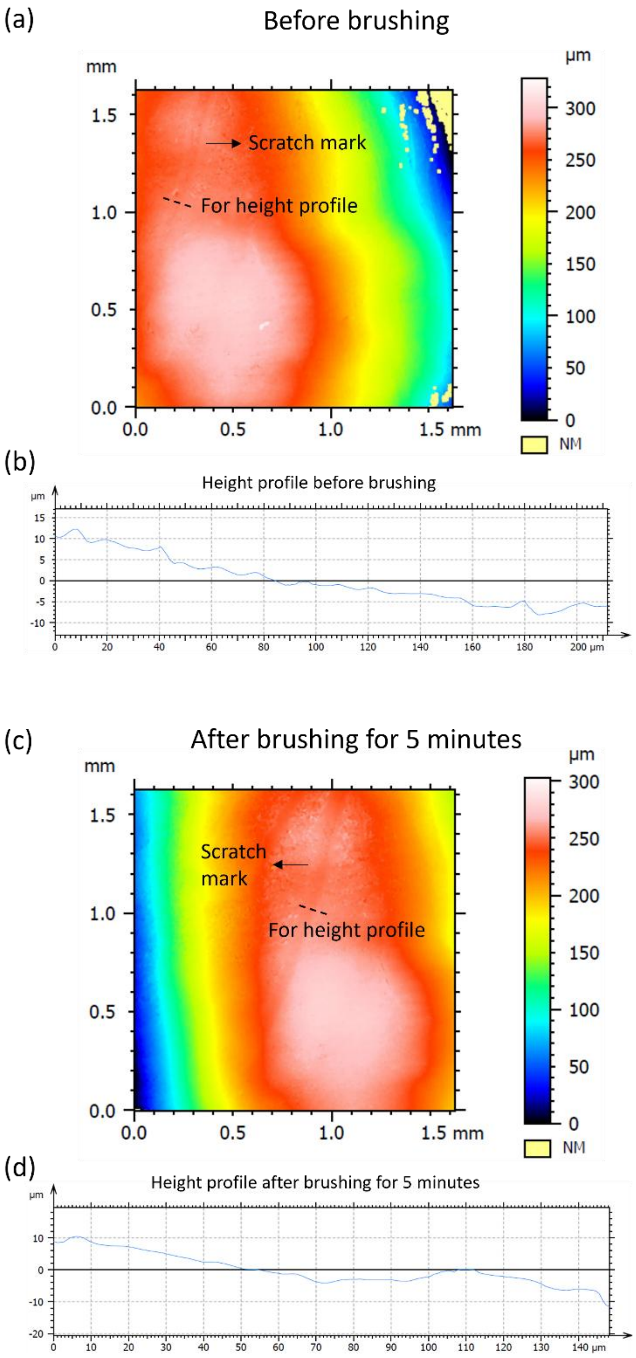


**Supplementary Fig. 51: Profilometry analysis of the ELR coated tooth enamel.**
**(a, c)** Heat maps of the ELR coated enamel before and after brushing for 5 mniutes. **(b, d)** Height profiles created across the scratch mark on the ELR coating before and after brushing for 5 mniutes.

**Discussion:** An ELR coating on an enamel surface was prepared using the ethanol/water solvent mixture and assessed for stability and bonding strength. Profilometry analysis revealed that there was no significant reduction in the ELR coating thickness after toothbrushing for 5 minutes (imitating 1 month of brushing), confirming its stability against physical wear.

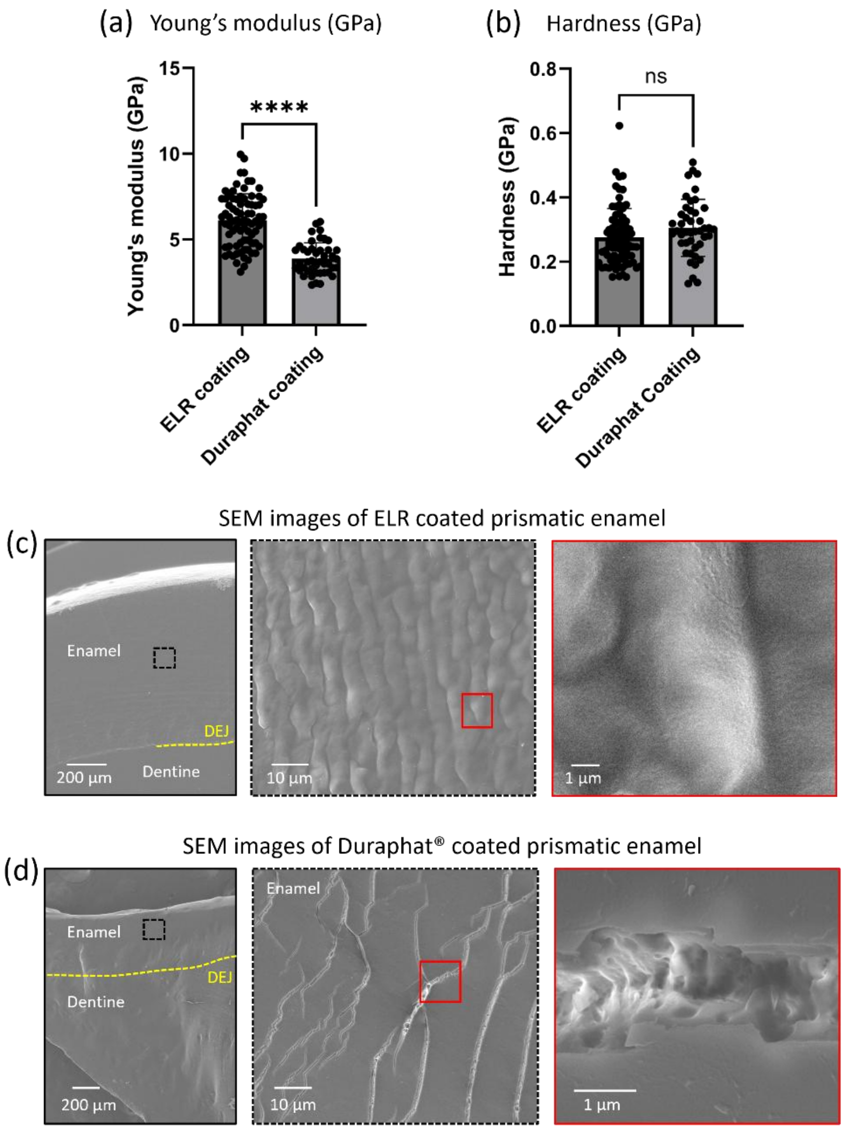


**Supplementary Fig. 52. ELR coating Vs Duraphat® coating.**

Comparison of the **(a)** Young's modulus (E) and **(b)** hardness (H) of the ELR and commercially available Duraphat® coating prepared on enamel surface. SEM images of enamel samples coated with **(c)** ELR and **(d)** Duraphat® coatings. Bars represent the mean values of E and H while dot plot represents indentation measurements compiled from 3 independent experiments for each group. Data are presented as mean ± SD. Statistical significance was analysed using two-tailed Welch's t-test in GraphPad Prism ver. 10. In **(a)** **** represents $p < 0.0001$, and in **(b)** 'ns' represents no significant difference.

**Supplementary references**


1 Elsharkawy, S. et al. Protein disorder–order interplay to guide the growth of hierarchical mineralized structures. *Nat. Commun.* **9**, 2145 (2018).

2 Lattanzi, V. et al. Amyloid β 42 fibril structure based on small-angle scattering. *Proc. Natl. Acad. Sci. USA* **118**, e2112783118 (2021).

3 Erickson, H. P. Size and shape of protein molecules at the nanometer level determined by sedimentation, gel filtration, and electron microscopy. *Biol. Proced. Online* **11**, 32-51 (2009).

4 Bromley, K. M. et al. Dissecting amelogenin protein nanospheres: characterization of metastable oligomers. *J. Biol. Chem.* **286**, 34643-34653 (2011).

5 Housmans, J. A., Wu, G., Schymkowitz, J. & Rousseau, F. A guide to studying protein aggregation. *FEBS J.* **290**, 554-583 (2023).

6 Beniash, E. et al. The hidden structure of human enamel. *Nat. Commun.* **10**, 4383 (2019).

7 Dal Sasso, G., Asscher, Y., Angelini, I., Nodari, L. & Artioli, G. A universal curve of apatite crystallinity for the assessment of bone integrity and preservation. *Sci. Rep.* **8**, 12025 (2018).

8 Pavese, M., Musso, S. & Pugno, N. M. Compression behaviour of thick vertically aligned carbon nanotube blocks. *J. Nanosci. Nanotechnol.* **10**, 4240-4245 (2010).

9 Lotsari, A., Rajasekharan, A. K., Halvarsson, M. & Andersson, M. Transformation of amorphous calcium phosphate to bone-like apatite. *Nat. Commun.* **9**, 4170 (2018).

10 Shao, C. et al. Repair of tooth enamel by a biomimetic mineralization frontier ensuring epitaxial growth. *Sci. Adv.* **5**, eaaw9569 (2019).

11 Fang, P.-A., Conway, J. F., Margolis, H. C., Simmer, J. P. & Beniash, E. Hierarchical self-assembly of amelogenin and the regulation of biomineralization at the nanoscale. *Proc. Natl. Acad. Sci. USA* **108**, 14097-14102 (2011).

12 Dey, A. et al. The role of prenucleation clusters in surface-induced calcium phosphate crystallization. *Nat. Mater.* **9**, 1010-1014 (2010).

13 Friddle, R. W. et al. Single-Molecule Determination of the Face-Specific Adsorption of Amelogenin's C-Terminus on Hydroxyapatite. *Angew. Chem. Int. Ed.* **123**, 7683-7687 (2011).

14 Lin, C. P., Douglas, W. H. & Erlandsen, S. L. Scanning electron microscopy of type I collagen at the dentin-enamel junction of human teeth. *J. Histochem. Cytochem.* **41**, 381-388 (1993).

15 Hayashi, Y. High resolution electron microscopy in the dentino-enamel junction. *J Electron Microsc.* **41**, 387-391 (1992).

16 Contessotto, P. et al. Elastin-like recombinamers-based hydrogel modulates post-ischemic remodeling in a non-transmural myocardial infarction in sheep. *Sci Transl. Med.* **13**, eaaz5380 (2021).

17 Ibáñez-Fonseca, A. et al. Elastin-like recombinamer hydrogels for improved skeletal muscle healing through modulation of macrophage polarization. *Front. Bioeng. Biotechnol.* **8**, 413 (2020).

18 Ibáñez-Fonseca, A. et al. Biocompatibility of two model elastin-like recombinamer-based hydrogels formed through physical or chemical cross-linking for various applications in tissue engineering and regenerative medicine. *J. Tissue Eng. Regen. Med.* **12**, 1450-1460 (2018).

19 Martinez-Avila, O. et al. Self-assembly of filamentous amelogenin requires calcium and phosphate: from dimers via nanoribbons to fibrils. *Biomacromolecules* **13**, 3494-3502 (2012).

20 Huang, J. & MacKerell Jr, A. D. CHARMM36 all-atom additive protein force field: Validation based on comparison to NMR data. *J. Comput. Chem.* **34**, 2135-2145 (2013).

21 Hub, J. S., De Groot, B. L. & van der Spoel, D. g_wham - A free weighted histogram analysis implementation including robust error and autocorrelation estimates. *J. Chem. Theory Comput.* **6**, 3713-3720 (2010).

22 Tejeda-Montes, E. et al. Bioactive membranes for bone regeneration applications: effect of physical and biomolecular signals on mesenchymal stem cell behavior. *Acta Biomater.* **10**, 134-141 (2014).

23 Tejeda-Montes, E. et al. Engineering membrane scaffolds with both physical and biomolecular signaling. *Acta Biomater.* **8**, 998-1009 (2012).
24 Tejeda-Montes, E. et al. Mineralization and bone regeneration using a bioactive elastin-like recombinamer membrane. *Biomater.* **35**, 8339-8347 (2014).
25 Ruben, J., Roeters, F., Montagner, A. & Huysmans, M. A multifunctional device to simulate oral ageing: the “Rub&Roll”. *J. Mech. Behav. Biomed. Mater.* **30**, 75-82 (2014).
26 Liu, X. et al. A comparison of in vitro cytotoxicity assays in medical device regulatory studies. *Regul. Toxicol. Pharmacol.* **97**, 24-32 (2018).
27 Wang, D. et al. Controlling enamel remineralization by amyloid-like amelogenin mimics. *Adv. Mater.* **32**, 2002080 (2020).
