# Supplementary material for: Biomimetic supramolecular protein matrix restores structure and properties of human dental enamel
Source: Nat Commun. 2025 Nov 4;16:9434. doi: 10.1038/s41467-025-64982-y (PMC12586552; doi:10.1038/s41467-025-64982-y)
Supplement: Supplementary file 2 — Reporting Summary [file 41467_2025_64982_MOESM2_ESM.pdf]

nature portfolio

Corresponding author(s): Alvaro Mata- NCOMMS-25-18400B

Last updated by author(s): Sep 27, 2025

Reporting Summary

Nature Portfolio wishes to improve the reproducibility of the work that we publish. This form provides structure for consistency and transparency in reporting. For further information on Nature Portfolio policies, see our Editorial Policies and the Editorial Policy Checklist.

Statistics

For all statistical analyses, confirm that the following items are present in the figure legend, table legend, main text, or Methods section.

n/a	Confirmed	
☐	☒	The exact sample size (*n*) for each experimental group/condition, given as a discrete number and unit of measurement
☐	☒	A statement on whether measurements were taken from distinct samples or whether the same sample was measured repeatedly
☐	☒	The statistical test(s) used AND whether they are one- or two-sided<br>*Only common tests should be described solely by name; describe more complex techniques in the Methods section.*
☐	☒	A description of all covariates tested
☒	☐	A description of any assumptions or corrections, such as tests of normality and adjustment for multiple comparisons
☐	☒	A full description of the statistical parameters including central tendency (e.g. means) or other basic estimates (e.g. regression coefficient) AND variation (e.g. standard deviation) or associated estimates of uncertainty (e.g. confidence intervals)
☒	☐	For null hypothesis testing, the test statistic (e.g. *F*, *t*, *r*) with confidence intervals, effect sizes, degrees of freedom and *P* value noted<br>*Give P values as exact values whenever suitable.*
☒	☐	For Bayesian analysis, information on the choice of priors and Markov chain Monte Carlo settings
☒	☐	For hierarchical and complex designs, identification of the appropriate level for tests and full reporting of outcomes
☒	☐	Estimates of effect sizes (e.g. Cohen's *d*, Pearson's *r*), indicating how they were calculated

Our web collection on statistics for biologists contains articles on many of the points above.

Software and code

Policy information about availability of computer code

Data collection	Device: infinite 200Pro-Tecan i-control , 2.0.10.0<br>Image J (Fiji) for TEM and Fluorescent microscopy<br>Cary 630, (Agilent, USA) for attenuated total reflection-Fourier-transform infrared (ATR-FTIR) spectroscopy analysis<br>MicroCal PEAQ-ITC (Malvern Instruments) for Isothermal titration calorimetry (ITC) analysis.<br>Leica Application Suite X (LAS X)
Data analysis	Stats and other graphs - GraphPad Prism v9.4.1 software (GraphPad Software, Inc).<br>Image J (Fiji) for fluorescence imaging and TEM analysis.<br>Gwyddion software (GNU General Public License) for atomic force microscope (AFM) analysis<br>GROMACS software (GNU Lesser General Public License) for molecular dynamics (MD) simulations<br>MARTINI 3 forcefield for coarse-grained MD simulations

For manuscripts utilizing custom algorithms or software that are central to the research but not yet described in published literature, software must be made available to editors and reviewers. We strongly encourage code deposition in a community repository (e.g. GitHub). See the Nature Portfolio guidelines for submitting code & software for further information.

Data

Policy information about availability of data

All manuscripts must include a data availability statement. This statement should provide the following information, where applicable:

- Accession codes, unique identifiers, or web links for publicly available datasets
- A description of any restrictions on data availability
- For clinical datasets or third party data, please ensure that the statement adheres to our policy

The data that support the findings of this study are provided in the Source Data file, Supplementary Information file, and are also available from the corresponding author upon request. Source data for all main and SI figures is available open access and can be found at DOI: 10.17639/nott.7600.

Research involving human participants, their data, or biological material

Policy information about studies with human participants or human data. See also policy information about sex, gender (identity/presentation), and sexual orientation and race, ethnicity and racism.

Reporting on sex and gender	N/A
Reporting on race, ethnicity, or other socially relevant groupings	N/A
Population characteristics	N/A
Recruitment	N/A
Ethics oversight	Human molar teeth extracted for clinical reasons were used in this study following approval from the Research Ethics Committee, Faculty of Medicine & Health Sciences, University of Nottingham (reference number: FMHS 313-0721). Informed consent was obtained from all donors prior to tooth extraction, with full protection of donor's privacy.

Note that full information on the approval of the study protocol must also be provided in the manuscript.

Field-specific reporting

Please select the one below that is the best fit for your research. If you are not sure, read the appropriate sections before making your selection.

☒ Life sciences ☐ Behavioural & social sciences ☐ Ecological, evolutionary & environmental sciences

For a reference copy of the document with all sections, see nature.com/documents/nr-reporting-summary-flat.pdf

Life sciences study design

All studies must disclose on these points even when the disclosure is negative.

Sample size	For in vitro studies, the sample size of each group was not less then 3.
Data exclusions	None excluded.
Replication	To verify reproducibility, a selection of tests was randomly chosen from the full set of experiments and assigned to a third person, who conducted them independently using standardized instructions. The resulting data were subsequently analyzed to confirm the consistency and reliability of the outcomes.
Randomization	Samples were randomly allocated into experiments group. For example, for enamel re-mineralization experiment, human teeth were sectioned into multiple sections and random sections were chosen for performing experiment.
Blinding	Investigators were blinded to the sample groups during analysis. Samples were labeled with alphanumeric codes prior to submission for evaluation—for example, before being sent to Nijmegen, Netherlands for Rub-n-Roll analysis (Chewing & grinding abrasion test), and to San Sebastian, Spain for TEM analysis.

Reporting for specific materials, systems and methods

We require information from authors about some types of materials, experimental systems and methods used in many studies. Here, indicate whether each material, system or method listed is relevant to your study. If you are not sure if a list item applies to your research, read the appropriate section before selecting a response.

Materials & experimental systems

n/a	Involved in the study
☒	☐ Antibodies
☐	☒ Eukaryotic cell lines
☒	☐ Palaeontology and archaeology
☒	☐ Animals and other organisms
☒	☐ Clinical data
☒	☐ Dual use research of concern
☒	☐ Plants

Methods

n/a	Involved in the study
☒	☐ ChIP-seq
☒	☐ Flow cytometry
☒	☐ MRI-based neuroimaging

Eukaryotic cell lines

Policy information about cell lines and Sex and Gender in Research

Cell line source(s)	NIH 3T3 fibroblasts (ATCC, USA)<br>Human immortalized mesenchymal stem cells (MSCs, Lonza, Switzerland)<br>Human umbilical vein endothelial cells (HUVECs, Lonza, Switzerland)
Authentication	None of the cells were authenticated.
Mycoplasma contamination	Cells were screened for mycoplasma on a monthly basis. Cells were cultured for one week in antibiotic-free medium before mycoplama testing. All lines were consistently confirmed to be mycoplasma-free.
Commonly misidentified lines<br>(See ICLAC register)	No commonly misidentified lines were used.

Plants

Seed stocks	N/A
Novel plant genotypes	N/A
Authentication	N/A
